# A Methodological and Survival Comparison of NCDB and SEER Database for Colon Cancer Research

**DOI:** 10.1002/jso.28141

**Published:** 2025-05-30

**Authors:** Metincan Erkaya, Ekin Inal, Cigdem Benlice, Mehmet Ayhan Kuzu, Emre Gorgun

**Affiliations:** ^1^ Department of Colorectal Surgery, Cleveland Clinic Digestive Disease and Surgery Institute Cleveland Ohio USA; ^2^ Department of General Surgery, School of Medicine Ankara University Ankara Turkey

**Keywords:** colon, colon cancer, colon cancer outcomes, National Cancer Database, NCDB, overall survival rates, SEER, Surveillance, Epidemiology, and End Results

## Abstract

**Background:**

The National Cancer Database (NCDB) and Surveillance, Epidemiology, and End Results (SEER) database are widely used in colon cancer research, particularly for analyzing overall survival (OS) rates. However, differences in demographics, treatment patterns, and survival outcomes across colon cancer stages and locations between these databases remain incompletely understood. Addressing these disparities is crucial for researchers when selecting the most appropriate registry for survival analysis.

**Objectives:**

This study aims to systematically compare patient characteristics, oncologic outcomes, and OS rates between NCDB and SEER across various tumor locations and disease stages in colon cancer.

**Methods:**

We analyzed patients undergoing surgery for Stages I–IV primary colon cancer (2004–2019), comparing patient characteristics, oncologic outcomes, and OS rates across distinct tumor locations and cancer stages in NCDB and SEER. Our objective was to assess how differences in database structure and sampling methodologies influence reported survival outcomes.

**Results:**

The study included 777 827 patients (NCDB: 572 196; SEER: 205 631). Proximal colon cancers were more common in older and female patients in both databases, whereas distal colon cancers were more prevalent in younger patients. NCDB contained a slightly higher proportion of Caucasian patients, while SEER had a greater representation of Asian patients. Segmental resections were more frequent in SEER, with the highest weighted difference observed in sigmoid colon cancer (6.16%; 95% CI: 5.78%–6.54%). OS rates were generally comparable across databases, though minor variations were observed at different colonic locations and stages.

**Conclusion:**

Despite differences in sampling techniques and follow‐up reporting, NCDB and SEER demonstrated remarkable consistency in survival trends across colon cancer stages and locations. Recognizing these database‐specific variations is essential for researchers conducting population‐based survival analyses and selecting the most suitable registry for their studies.

**What does this paper add to the literature?:**

This study provides a comprehensive comparison of NCDB and SEER, highlighting how differences in sampling methodologies, follow‐up reporting, and patient representation influence overall survival estimates in colon cancer. It clarifies why prior studies report conflicting survival trends and offers a methodological framework for researchers selecting the most appropriate database for their analysis.

## 1 Introduction

The population‐based evaluation of oncologic outcomes is a crucial indicator of curative cancer treatment. While individual hospitals maintain their own databases, national cancer databases provide a broader and more representative assessment of cancer care. Two widely used databases in colon cancer research are the National Cancer Database (NCDB) and the Surveillance, Epidemiology, and End Results (SEER) database. These databases differ in scope and methodology, as NCDB is a hospital‐based registry, while SEER is a population‐based registry. Previous studies have compared these databases in terms of patient demographics, tumor characteristics, and treatment variables across various cancer types [1, 2, 3], showing some degree of similarity. However, colon cancer presents methodological challenges due to its high variability in tumor location (right vs. left vs. transverse), stage‐dependent treatment approaches, and evolving surgical and systemic therapy paradigms. These factors may contribute to significant differences in survival estimates when using NCDB versus SEER, which have not been systematically evaluated.

Prior studies utilizing NCDB and SEER for colon cancer research have reported conflicting overall survival (OS) trends, particularly regarding differences in tumor location and stage‐specific outcomes. NCDB‐based studies have suggested that stage I and II left‐sided colon cancers exhibit poorer survival rates compared to right‐sided colon cancers, whereas SEER‐based studies have reported mixed findings, with disagreements on which tumor locations and stages confer a survival advantage [4, 5, 6, 7, 8]. The choice of database may substantially impact research conclusions, not necessarily due to true differences in clinical outcomes, but rather due to structural methodological biases. NCDB, which captures data from CoC‐accredited hospitals, may introduce selection bias favoring patients with better access to specialized cancer care. In contrast, SEER, which relies on state registries and national death certificates, provides more comprehensive mortality data but lacks some detailed treatment‐related variables that are crucial for evaluating perioperative and oncologic outcomes.

Recognizing these database‐specific differences is essential for ensuring valid survival comparisons and selecting the most appropriate dataset for research. However, despite the frequent use of NCDB and SEER in colon cancer studies, the potential disparities in patient demographics, treatment patterns, and survival outcomes across tumor locations and stages remain insufficiently explored. This study aims to compare NCDB and SEER in terms of patient characteristics, oncologic outcomes, and survival data for colon cancer across various stages and anatomical locations. We hypothesize that NCDB and SEER report similar survival outcomes, but variations in sampling methods may contribute to differences seen in prior studies. Our results will provide a methodological framework to guide researchers in selecting the most suitable database for colon cancer research.

## 2 Materials and Methods

### 2.1 Study Cohort

**Figure 1:**
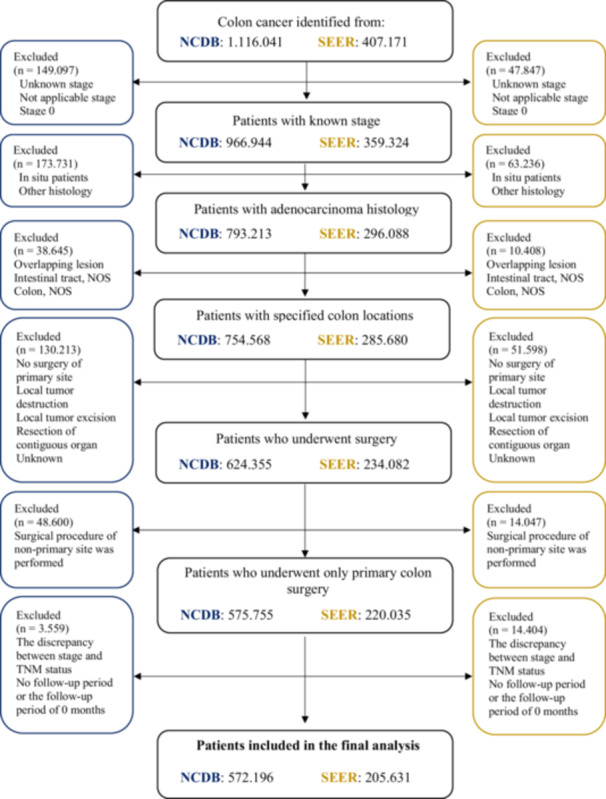
NCDB and SEER database flow chart.

Data were obtained from the NCDB and SEER database from 2004 to 2019. Patients with primary colon cancer were identified using International Classification of Diseases for Oncology topographic codes, ranging from C18.0 (cecum) to C18.7 (sigmoid colon). Patients with adenocarcinoma, as defined by the histologic codes 8140, 8210‐11, 8260, 8262, and 8263, were included in this study. A total of 1 523 212 primary colon cancer patients (NCDB: 1 116 041; SEER: 407 171) were identified. Patients in the final study were chosen based on our inclusion criteria, including known stage, adenocarcinoma histology, distinct colonic cancer sites, and patients who underwent surgery. Overall, 777 827 patients (NCDB: 572 196; SEER: 205 631) met our inclusion criteria, as shown in Figure 1. Considering that de‐identified patient data obtained from the NCDB and SEER database are lawfully accessible to the general public, ethical approval was not required.

### 2.2 NCDB

The National Cancer Database is a comprehensive clinical oncology database with data from hospital registries across the United States. It is one of the largest cancer registries in the country, encompassing nearly 34 million cases from over 1500 hospitals. The NCDB is a collaborative project between the American Cancer Society and the Commission on Cancer (CoC) of the American College of Surgeons. Its primary purpose is surveillance and quality improvement in cancer care, capturing approximately 72% of all newly diagnosed malignancies in the United States [9].

### 2.3 SEER Database

SEER database is a population‐based cancer registry that covers various regions of the United States. It is an initiative of the National Cancer Institute and aims to provide information on cancer incidence and survival in the country. SEER database collects and shares data on cancer from registries that cover around 48% of the U.S. population. SEER database is population‐based, allowing for the calculation of cancer incidence. It collects comprehensive demographic, clinical, and outcome information on all cancers diagnosed in representative geographic regions and subpopulations reflective of the U.S. census [10].

### 2.4 Variable Differences in NCDB and SEER Database

**Figure 2:**
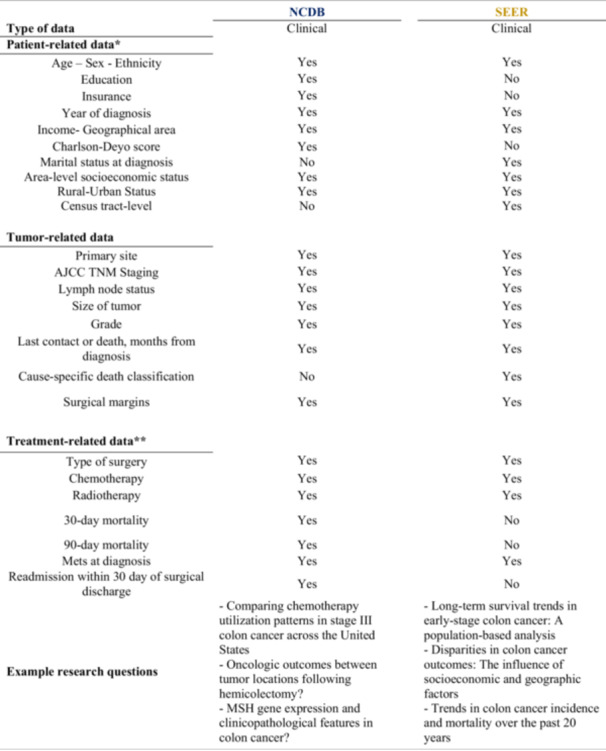
Comparison of variables included in NCDB and SEER database. *Additional, more detailed information available in the SEER database **NCDB has more subvariables in treatment‐related data compared to SEER.

The NCDB and SEER databases differ in data collection methods, scope, and available variables, influencing their applicability in colon cancer research. While some variables are shared between both databases, others are unique to each, impacting the interpretation of oncologic outcomes. These differences are summarized in Figure 2.

- ***Sociodemographic Variables:*** Both databases include age at diagnosis, sex, race/ethnicity, and geographic location. However, SEER provides more detailed socioeconomic data at the county and census‐tract levels, including education level, poverty rate, and rural‐urban classification. In contrast, NCDB reports ZIP Code‐level socioeconomic indicators and includes data on patient distance to treatment facilities.
- ***Clinical and Tumor Characteristics:*** Both databases capture tumor histology, grade, and staging information (AJCC TNM classification). However, NCDB includes the Charlson‐Deyo comorbidity score, which is not available in SEER. SEER uniquely tracks cancer incidence and cause‐specific mortality, whereas NCDB provides additional data on treatment facility characteristics and hospital‐specific outcomes. Both databases contain a few staging variables, but we focused on those that prioritize pathologic staging, with clinical staging used when pathologic data is unavailable. To ensure accuracy, we included only patients who underwent surgery and excluded cases with staging inconsistencies separately, minimizing discrepancies and enhancing comparability between NCDB and SEER.
- ***Treatment Variables:*** Both databases contain information on surgical procedures, chemotherapy, and radiation therapy. However, NCDB provides more treatment details, including surgical approach (open, minimally invasive, robotic), surgical margins, time from diagnosis to first treatment, radiation dose and fractionation, reasons for withholding radiation or chemotherapy, and receipt of hormone therapy, immunotherapy, or palliative care. SEER lacks detailed chemotherapy and radiation therapy information and does not track postoperative complications or readmission rates, whereas NCDB includes 30‐ and 90‐day postoperative mortality.
- ***Follow‐up and Survival Data:*** SEER determines vital status through national mortality data and state registries, allowing for cause‐specific survival analysis. NCDB relies on hospital‐reported follow‐up data, which may introduce variability in survival reporting. Follow‐up time is available in both datasets and is calculated from the date of diagnosis to the date of death (SEER and NCDB), date of last contact (NCDB), or date last presumed alive (SEER), reported in months.

### 2.5 Outcome Measures

The primary outcome was the comparison of OS across distinct colon cancer locations between NCDB and SEER. Subgroup analyses were conducted for Stages I–IV to identify potential discrepancies in OS rates. Due to the lack of cancer‐specific survival data in NCDB, OS was used as a comprehensive measure of long‐term outcomes. Secondary outcomes included weighted differences in demographics, tumor characteristics, and treatment variables.

### 2.6 Statistical Analysis

Given the variance in sample size between the NCDB and SEER databases, weighted proportional differences were used to compare categorical variables while accounting for differences in sample size distribution. This method helps mitigate biases introduced by unequal sample sizes and ensures a balanced comparison between the two databases. 95% confidence intervals (CIs) were computed to assess statistical significance, where a difference was considered significant if the CI did not cross zero.

This approach was favored over traditional *p*‐values because *p*‐values are highly sensitive to large sample sizes, potentially detecting statistically significant differences that are not clinically meaningful. Additionally, weighted proportional differences help address potential overlap of patients between the two databases, reducing the risk of interdependent observations affecting statistical outcomes [11, 12].

For OS analysis, Kaplan–Meier (KM) survival estimates were used to compare survival distributions between NCDB and SEER across different tumor locations and stages (I–IV). The log‐rank test was performed to determine statistical differences in survival curves. To further explore differences in OS, median survival times and 5‐year OS rates were calculated and reported with 95% CIs. To enable a more precise and direct quantitative comparison of survival differences, OS estimates from KM curves were numerically extracted at 1‐, 3‐, 5‐, and 10‐year time points. Subgroup analyses were conducted to examine OS trends across specific tumor locations and staging categories, allowing for a more refined understanding of potential database discrepancies. All statistical analyses were conducted using R version 4.2.3.

## 3 Results

A total of 777 827 patients were included, with 572 196 (73.6%) from NCDB and 205 631 (26.4%) from SEER. The mean (SD) age was 68.5 (13.1) years in NCDB and 67.9 (12.7) years in SEER. The sex distribution was similar in both databases, with NCDB: 50.4% female, 49.6% male and SEER: 49.7% female, 50.3% male. Demographic patterns showed that proximal colon cancers (cecum, ascending, hepatic flexure, and transverse colon) were associated with older age and a higher proportion of female patients, whereas distal colon cancers (descending, sigmoid) had a younger patient population. Appendiceal cancer patients were younger and predominantly male. The median follow‐up time was 55 months (IQR: 24–104) in the SEER database and 51 months (IQR: 24–94) in the NCDB.

Beyond these general patterns, notable differences in patient characteristics between NCDB and SEER were observed. Several demographic characteristics showed significant proportional differences between the databases. Caucasian patients were more frequently recorded in NCDB across all colon cancer locations, with a notable difference in the sigmoid colon. Conversely, the “Other” racial category (primarily Asian and some American Indian) was more common in SEER, with the highest weighted difference observed in the sigmoid colon (6.3%; 95% CI: 6.02%–6.59%). Demographic and patient characteristics across colonic cancer locations in NCDB and SEER are detailed in Tables 1, 2, 3. A higher proportion of patients in SEER resided in areas with a population of 1 million or more (57.6%) compared to NCDB (51.6%), as shown in the heatmap (Supporting Information S6: Figure S5).

**Table 1:** Demographics and patient characteristics (right‐sided colon cancer).

Variable	Cecum			Ascending colon			Hepatic flexure of colon		
	NCDB *N* = 132.376	SEER *N* = 47.247	CI^a^	NCDB *N* = 129.473	SEER *N* = 43.846	CI^a^	NCDB *N* = 28.175	SEER *N* = 10.466	CI^a^
Age, *n* (%)									
18–49 years	7.631 (5.77)	2.636 (5.58)	0.18% (−0.05 to 0.42)	7.144 (5.52)	2.527 (5.77)	0.25% (−0.01 to 0.49)	1.782 (6.32)	707 (6.75)	0.43% (−0.12 to 0.98)
50–64 years	30.489 (23.02)	11.031 (23.36)	0.31% (−0.12 to 0.75)	29.503 (22.80)	10.283 (23.47)	0.66% (0.21 to 1.12)	6.509 (23.11)	2.502 (23.91)	0.8% (−0.14 to 1.75)
65–79 years	55.694 (42.08)	19.976 (42.28)	0.2% (−0.06 to 0.47)	56.506 (43.66)	19.003 (43.34)	0.3% (0.03 to 0.58)	12.341 (43.85)	4.494 (42.92)	0.86% (0.25 to 1.46)
80+	38.562 (29.13)	13.604 (28.78)	0.33% (−0.13 to 0.81)	36.320 (28.02)	12.033 (27.42)	0.6% (0.12 to 1.09)	7.543 (26.72)	2.763 (26.42)	0.37% (−0.61 to 1.36)
Age (years), mean (SD)	70.98 (±12.56)	70.55 (±11.98)	0.04	70.90 (±12.36)	70.25 (±11.99)	0.05	70.41 (±12.60)	69.72 (±12.33)	0.06
Age (years), median (interquartile range)	73 (18)	72 (18)		72 (18)	72 (17)		72 (18)	72 (18)	
Gender, *n* (%)									
Male (%)	59.035 (44.6)	21.330 (45.1)	0.55% (0.03 to 1.07)	59.466 (45.99)	20.498 (46.77)	0.82% (0.28 to 1.36)	14.233 (50.53)	5.355 (51.16)	0.65% (−0.47 to 1.77)
Female (%)	73.341 (55.4)	25.917 (54.9)		70.007 (54.01)	23.348 (53.23)		13.942 (49.47)	5.111 (48.84)	
Ethnicity, *n* (%)									
Caucasian	111.664 (84.2)	38.545 (81.6)	2.77% (2.37 to 3.17)	108.538 (83.87)	35.171 (80.22)	3.61% (3.19 to 4.01)	23.860 (84.71)	8.455 (80.70)	3.89% (3.03 to 4.76)
African–American	16.008 (12.2)	5.695 (12.1)	0.04% (−0.3 to 0.38)	15.459 (11.94)	5.255 (11.99)	0.04% (−0.3 to 0.39)	3.063 (10.86)	1.113 (10.63)	0.23% (−0.45 to 0.93)
Other	3.808 (2.9)	2.867 (5.9)	3.19% (2.95 to 3.42)	4.519 (3.47)	3.271 (7.46)	3.96% (3.7 to 4.23)	1.054 (3.73)	865 (8.25)	4.52% (3.95 to 5.09)
Unknown	896 (0.7)	140 (0.4)	0.38% (0.31 to 0.44)	957 (0.72)	149 (0.33)	0.39% (0.32 to 0.47)	198 (0.70)	33 (0.33)	0.38% (0.24 to 0.53)
Charlson‐Deyo score, *n* (%)									
0	87.981 (66.4)	—		84.331 (65.16)	—		18120 (64.29)	—	
1	29.218 (22.1)	—		29.278 (22.60)	—		6551 (23.25)	—	
2	9.608 (7.3)	—		9.932 (7.66)	—		2226 (7.91)	—	
3+	5.569 (4.2)	—		5.932 (4.58)	—		1278 (4.55)	—	
Marital status at diagnosis, *n* (%)									
Divorced	—	4.181 (8.84)		—	3751 (8.56)		—	892 (8.52)	
Married (including common law)	—	24.367 (51.60)		—	23.237 (53.05)		—	5717 (54.55)	
Separated	—	410 (0.87)		—	371 (0.84)		—	112 (1.09)	
Single (never married)	—	6.110 (12.92)		—	5.437 (12.37)		—	1291 (12.34)	
Unmarried or domestic partner	—	76 (0.16)		—	55 (0.12)		—	17 (0.18)	
Widowed	—	9.977 (21.11)		—	9.038 (20.60)		—	1984 (18.97)	
Unknown	—	2.126 (4.50)		—	1.957 (4.46)		—	453 (4.35)	

Abbreviations: CI, confidence interval; NCDB, National Cancer Database; NOS, not otherwise specified; SD, standard deviation; SEER, Surveillance, Epidemiology, and End Results.

^a^ Weighted‐proportional difference/standardized difference (95% CI).

**Table 2:** Demographics and patient characteristics (left‐sided colon cancer).

Variable	Splenic flexure of colon			Descending colon			Sigmoid colon		
	NCDB *N* = 19.407	SEER *N* = 7.232	CI^a^	NCDB *N* = 37.744	SEER *N* = 13.557	CI^a^	NCDB *N* = 161.492	SEER *N* = 60.581	CI^a^
Age, *n* (%)									
18–49 years	1.982 (10.22)	731 (10.10)	0.1% (−0.71 to 0.91)	4.549 (12.05)	1.666 (12.28)	0.23% (−0.4 to 0.87)	20.711 (12.82)	7.622 (12.57)	0.24% (−0.06 to 0.55)
50–64 years	5.720 (29.43)	2.226 (30.78)	1.3% (0.06 to 2.54)	12.305 (32.61)	4.447 (32.81)	0.2% (−0.71 to 1.12)	58.222 (36.03)	22.470 (37.09)	1.03% (−0.58 to 1.48)
65–79 years	7.659 (39.44)	2.815 (38.94)	0.54% (−0.16 to 1.24)	14.348 (38.01)	5.156 (38.03)	0.01% (−0.47 to 0.5)	58.498 (36.23)	21.826 (36.01)	0.19% (−0.04 to 0.43)
80+	4.046 (20.91)	1.460 (20.18)	0.66% (−0.42 to 1.74)	6.542 (17.33)	2.288 (16.88)	0.45% (−0.28 to 1.19)	24.061 (14.92)	8.663 (14.33)	0.59% (0.27 to 0.92)
Age (years), mean (standard deviation)	67.30 (±13.49)	66.72 (±13.09)	0.04	65.72 (±13.53)	65.27 (±13.25)	0.03	64.59 (±13.28)	64.23 (±12.83)	0.03
Age (years), median (interquartile range)	68 (20)	68 (19)		66 (20)	66 (20)		65 (20)	65 (19)	
Gender, *n* (%)									
Male (%)	10.538 (54.27)	3.985 (55.14)	0.8% (−0.54 to 2.14)	20.401 (54.05)	7.417 (54.75)	0.65% (−0.31 to 1.63)	88.973 (55.10)	33.506 (55.26)	0.21% (−0.25 to 0.67)
Female (%)	8.869 (45.73)	3.247 (44.86)		17.343 (45.95)	6.140 (45.25)		72.519 (44.90)	27.075 (44.74)	
Ethnicity, *n* (%)									
Caucasian	15.421 (79.45)	5.462 (75.58)	3.93% (2.79 to 5.07)	29.686 (78.68)	10.004 (73.80)	4.85% (4.01 to 5.7)	133.899 (82.89)	46.962 (77.53)	5.39% (5.01 to 5.77)
African–American	3.061 (15.76)	1.079 (14.92)	0.85% (−0.11 to 1.82)	5.790 (15.33)	1.944 (14.32)	1.0% (0.3 to 1.69)	16.609 (10.29)	5.899 (9.74)	0.54% (0.26 to 0.82)
Other	797 (4.11)	663 (9.18)	5.06% (4.33 to 5.78)	1.958 (5.17)	1.560 (11.52)	6.31% (5.73 to 6.9)	9.583 (5.94)	7.417 (12.23)	6.3% (6.02 to 6.59)
Unknown	128 (0.66)	28 (0.39)	0.27% (0.08 to 0.45)	310 (0.82)	49 (0.36)	0.45% (0.32 to 0.59)	1.401 (0.88)	303 (0.50)	0.36% (0.29 to 0.43)
Charlson‐Deyo score, *n* (%)									
0	13.137 (67.56)	—		26.185 (69.31)	—		115.046 (71.20)	—	
1	4.261 (21.92)	—		7.836 (20.73)	—		32.251 (19.98)	—	
2	1.295 (6.66)	—		2.285 (6.15)	—		9.118 (5.66)	—	
3+	714 (3.67)	—		1.438 (3.81)	—		5.077 (3.16)	—	
Marital status at diagnosis, *n* (%)									
Divorced	—	701 (9.70)		—	1.236 (9.11)		—	5.504 (9.09)	
Married (including common law)	—	3.892 (53.80)		—	7.497 (55.34)		—	34.539 (57.03)	
Separated	—	66 (0.91)		—	130 (0.95)		—	629 (1.04)	
Single (never married)	—	1.142 (15.80)		—	2.130 (15.70)		—	9.337 (15.41)	
Unmarried or domestic partner	—	13 (0.18)		—	27 (0.20)		—	143 (0.23)	
Widowed	—	1.138 (15.74)		—	1.961 (14.46)		—	7.697 (12.70)	
Unknown	—	280 (3.87)		—	576 (4.24)		—	2.732 (4.50)	

Abbreviations: CI, confidence interval; NCDB, National Cancer Database; NOS, not otherwise specified; SD, standard deviation; SEER, Surveillance, Epidemiology, and End Results.

^a^ Weighted‐proportional difference/standardized difference (95% CI).

**Table 3:** Demographics and patient characteristics (other colon locations).

Variable	Transverse colon			Appendix		
	NCDB *N* = 59.406	SEER *N* = 21.239	CI^a^	NCDB *N* = 4.123	SEER *N* = 1.463	CI^a^
Age, *n* (%)						
18–49 years	4.154 (6.99)	1.586 (7.48)	0.47% (0.06 to 0.88)	659 (15.98)	236 (16.13)	0.14% (−2.04 to 2.33)
50–64 years	14.350 (24.16)	5.330 (25.10)	0.93% (0.26 to 1.61)	1.427 (34.61)	510 (34.86)	0.24% (−2.59 to 3.09)
65–79 years	25.055 (42.21)	8.809 (41.42)	0.7% (0.28 to 1.11)	1.481 (35.92)	507 (34.66)	1.26% (−0.3 to 2.83)
80+	15.847 (26.64)	5.514 (26.00)	0.71% (0.02 to 1.4)	556 (13.49)	210 (14.35)	0.86% (−1.2 to 2.94)
Age (years), mean (standard deviation)	70.0 (±12.96)	69.28 (±12.56)	0.06	63.45 (±14.08)	63.3 (±13.78)	0.01
Age (years), median (interquartile range)	72 (18)	71 (19)		64 (20)	64 (20)	
Gender, *n* (%)						
Male (%)	28.884 (48.57)	10.531 (49.61)	0.96% (0.17 to 1.74)	2.319 (56.25)	813 (55.57)	0.67% (−2.2 to 3.63)
Female (%)	30.522 (51.43)	10.708 (50.39)		1.804 (43.75)	650 (44.43)	
Ethnicity, *n* (%)						
Caucasian	49.938 (83.98)	16.975 (79.87)	4.13% (3.52 to 4.75)	3.409 (82.68)	1.148 (78.47)	4.21% (1.81 to 6.61)
African–American	6.869 (11.58)	2.485 (11.72)	0.13% (−0.36 to 0.64)	536 (13.0)	209 (14.29)	1.28% (−0.78 to 3.35)
Other	2.179 (3.70)	1.717 (8.10)	4.41% (4.01 to 4.81)	147 (3.57)	101 (6.9)	3.33% (1.92 to 4.75)
Unknown	420 (0.74)	62 (0.31)	0.41% (0.31 to 0.51)	31 (0.75)	5 (0.34)	0.41% (0.01 to 0.8)
Charlson‐Deyo score, *n* (%)						
0	38.693 (65.13)	—		3.047 (73.90)	—	
1	13.241 (22.28)	—		713 (17.29)	—	
2	4.584 (7.71)	—		226 (5.48)	—	
3+	2.888 (4.86)	—		137 (3.33)	—	
Marital status at diagnosis, *n* (%)						
Divorced	—	1.849 (8.70)		—	133 (9.09)	
Married (including common law)	—	11.343 (53.40)		—	850 (58.1)	
Separated	—	191 (0.91)		—	11 (0.75)	
Single (never married)	—	2.746 (12.92)		—	248 (16.95)	
Unmarried or domestic partner	—	30 (0.14)		—	3 (0.21)	
Widowed	—	4164 (19.61)		—	162 (11.07)	
Unknown	—	916 (4.32)		—	56 (3.83)	

Abbreviations: CI, confidence interval; NCDB, National Cancer Database; NOS, not otherwise specified; SD, standard deviation; SEER, Surveillance, Epidemiology, and End Results.

^a^ Weighted‐proportional difference/standardized difference (95% CI).

Differences were also observed in surgical management. Segmental resections were more frequent in SEER, with the highest weighted difference observed in sigmoid colon cancer (6.16%; 95% CI: 5.78%–6.54%). For lymph node retrieval, NCDB reported a higher proportion of cases with ≥ 17 lymph nodes retrieved, although these differences were minimal across tumor locations. Conversely, SEER had a higher proportion of cases with < 12 lymph nodes retrieved, with the largest difference observed in appendix cancer (6.55%; 95% CI: 3.97%–9.14%). Chemotherapy use was slightly higher in NCDB across tumor locations, with a weighted difference of 3.7% (95% CI: 3.25%–4.16%) for sigmoid colon cancer and 4.05% (95% CI: 1.17%–6.93%) for appendix cancer. Tumor characteristics are detailed in Tables 4, 5, 6.

**Table 4:** Tumor and treatment characteristics of primary colon cancer (right‐sided colon cancer).

Variable	Cecum			Ascending colon			Hepatic flexure of colon		
	NCDB *N* = 132.376	SEER *N* = 47.247	CI^a^	NCDB *N* = 129.473	SEER *N* = 43.846	CI^a^	NCDB *N* = 28.175	SEER *N* = 10.466	CI^a^
Tumor stage, *n* (%)									
Stage I	34.414 (25.98)	12.415 (26.28)	0.28% (−0.18 to 0.74)	34.737 (26.81)	11.974 (27.28)	0.48% (−0.002 to 0.96)	6.994 (24.82)	2.559 (24.45)	0.37% (−0.59 to 1.33)
Stage II	40.461 (30.56)	14.694 (31.1)	0.54% (0.05 to 1.02)	45.531 (35.17)	15.573 (35.54)	0.35% (−0.16 to 0.86)	10.745 (38.17)	3.984 (38.05)	0.07% (−1.01 to 1.16)
Stage III	44.130 (33.38)	15.073 (31.9)	1.43% (0.94 to 1.92)	39.194 (30.30)	12.781 (29.13)	1.12% (0.62 to 1.61)	8.167 (28.98)	3.008 (28.73)	0.24% (−0.77 to 1.26)
Stage IV	13.371 (10.08)	5.065 (10.72)	0.62% (0.29 to 0.94)	10.011 (7.72)	3.518 (8.05)	0.29% (−0.001 to 0.58)	2.269 (8.03)	915 (8.77)	0.68% (0.06 to 1.31)
Grade, *n* (%)									
G1	12.746 (9.62)	3.977 (8.42)	1.21% (0.91 to 1.51)	12.863 (9.94)	3.826 (8.71)	1.21% (0.89 to 1.52)	2.483 (8.80)	781 (7.47)	1.35% (0.74 to 1.95)
G2	87.793 (66.34)	32.225 (68.14)	1.88% (1.39 to 2.38)	84.903 (65.56)	29.592 (67.54)	1.91% (1.40 to 2.42)	18.715 (66.46)	7.145 (68.25)	1.84% (0.79 to 2.82)
G3	25.197 (19.04)	8.820 (18.69)	0.36% (−0.04 to 0.78)	24.739 (19.12)	8.131 (18.52)	0.56% (0.14 to 0.98)	5.396 (19.15)	2.003 (19.13)	0.01% (−0.86 to 0.89)
G4	3.070 (2.31)	1.180 (2.52)	0.17% (0.02 to 0.34)	2.875 (2.22)	1.100 (2.50)	0.28% (0.12 to 0.45)	659 (2.33)	244 (2.34)	0.007% (−0.33 to 0.34)
Unknown	3.570 (2.69)	1.045 (2.23)	0.48% (0.32 to 0.64)	4.093 (3.16)	1.197 (2.73)	0.43% (0.25 to 0.61)	922 (3.26)	293 (2.81)	0.47% (0.09 to 0.85)
T‐stage, *n* (%)									
T1	15.098 (11.38)	5.867 (12.41)	1.01% (0.67 to 1.36)	17.194 (13.28)	6.613 (15.09)	1.8% (1.41 to 2.18)	3.338 (11.83)	1.297 (12.40)	0.54% (−0.19 to 1.28)
T2	24.138 (18.22)	8.800 (18.62)	1.91% (−0.01 to 0.8)	20.817 (16.09)	7.036 (16.04)	0.03% (−0.36 to 0.42)	4.142 (14.70)	1.595 (15.26)	0.53% (−0.26 to 1.34)
T3	66.300 (50.13)	24.768 (52.42)	2.33% (1.81 to 2.86)	71.917 (55.54)	25.333 (57.76)	2.23% (1.69 to 2.76)	16.295 (57.87)	6.363 (60.71)	2.96% (1.86 to 4.06)
T4	20.910 (15.80)	7.655 (16.21)	0.40% (0.02 to 0.79)	13.545 (10.46)	4.662 (10.64)	0.17% (−0.16 to 0.5)	3.010 (10.67)	1.160 (11.11)	0.4% (−0.3 to 1.1
Unknown	5.930 (4.47)	157 (0.34)	4.14% (4.02 to 4.27)	6.000 (4.63)	202 (0.47)	4.17% (4.04 to 4.3)	1.390 (4.93)	51 (0.52)	4.44% (4.16 to 4.73)
Number of retrieved regional lymph node, *n* (%)									
< 12 lymph nodes	7.330 (5.53)	3.159 (6.69)	1.14% (0.89 to 1.41)	5.922 (4.56)	2532 (5.78)	1.2% (0.95 to 1.44)	1.416 (5.02)	642 (6.15)	1.1% (0.58 to 1.63)
12–16 lymph nodes	40.333 (30.48)	14.673 (31.06)	0.59% (0.10 to 1.07)	35.001 (27.05)	12.039 (27.47)	0.42% (−0.05 to 0.9)	7.226 (25.65)	2.790 (26.65)	1.01% (0.02 to 1.99)
17+ lymph nodes	84.151 (63.58)	29.135 (61.64)	1.90% (1.40 to 2.41)	88.022 (67.99)	29.054 (66.23)	1.72% (1.21 to 2.23)	19.420 (68.94)	6.973 (66.59)	2.3% (1.24 to 3.35)
Unknown/missing	562 (0.41)	280 (0.61)	0.16% (0.09 to 0.24)	528 (0.40)	221 (0.52)	0.09% (0.02 to 0.17)	113 (0.39)	61 (0.61)	0.18% (0.01 to 0.34)
Mean (SD)	19.49 (±9.64)	18.69 (±9.47)	0.08	20.87 (±10.36)	20.09 (±10.12)	0.08	21.20 (±11.11)	20.32 (±10.92)	0.09
0	76.794 (58.02)	27.805 (58.80)	0.83% (0.32 to 1.35)	82.244 (63.53)	28.197 (64.26)	0.78% (0.26 to 1.3)	18.302 (64.95)	6.758 (64.53)	0.38% (−0.68 to 1.45)
1	14.502 (10.95)	4.896 (10.36)	0.59% (0.27 to 0.91)	14.141 (10.92)	4.654 (10.62)	0.3% (−0.03 to 0.64)	3.299 (11.71)	1.184 (11.31)	0.39% (−0.31 to 1.1)
2–3	16.282 (12.29)	5.724 (12.12)	0.18% (−0.15 to 0.52)	14.551 (11.25)	4.735 (10.82)	0.43% (0.1 to 0.77)	3.091 (10.97)	1.157 (11.05)	0.08% (−0.61 to 0.78)
4–6	11.819 (8.93)	4.083 (8.65)	0.28% (−0.01 to 0.58)	9.282 (7.16)	3.043 (6.95)	0.22% (−0.05 to 0.5)	1.760 (6.24)	711 (6.81)	0.54% (−0.01 to 1.1)
7+	5.926 (4.48)	2.090 (4.45)	0.16% (0.09 to 0.24)	4.069 (3.14)	1.345 (3.08)	0.08% (−0.11 to 0.26)	791 (2.81)	258 (2.48)	0.34% (−0.01 to 0.69)
Unknown/missing	7.053 (5.33)	2.649 (5.62)	0.27% (0.04 to 0.52)	5.186 (4.00)	1.872 (4.27)	0.26% (0.04 to 0.48)	932 (3.32)	398 (3.82)	0.49% (0.07 to 0.91)
Mean (SD)	1.93 (±3.83)	1.88 (±3.73)	0.01	1.53 (±3.49)	1.45 (±3.30)	0.02	1.32 (±3.16)	1.29 (±2.95)	0.01
Tumor size (mm), mean (SD)	50.94 (±58.26)	48.57 (±36.14)	0.05	48.83 (±62.64)	46.07 (±34.75)	0.05	47.22 (±55.16)	45.78 (±35.53)	0.03
Tumor size (mm), median (interquartile range)	45 (30)	45 (30)		41 (32)	40 (32)		40 (32)	40 (31)	
First surgical procedure time, months from diagnosis, mean [range–day]	0.58 [0–660]	0.56 [0–660]		0.60 [0–660]	0.59 [0–660]		0.64 [0–651]	0.62 [0–570]	
Vital status, *n* (%)									
Alive	67.438 (50.91)	22.600 (47.78)	3.11% (2.59 to 3.64)	69.277 (53.49)	22.646 (51.63)	1.86% (1.32 to 2.4)	14.611 (51.82)	5.140 (49.03)	2.74% (1.62 to 3.86)
Dead	64.938 (49.09)	24.647 (52.22)		60.196 (46.51)	21.200 (48.37)		13.564 (48.18)	5.326 (50.97)	
Chemotherapy, *n* (%)									
Yes	43.405 (32.77)	14.160 (29.92)	2.82% (2.33 to 3.30)	37.541 (29.02)	11.608 (26.51)	2.52% (2.04 to 3.0)	8.296 (29.45)	2.973 (28.36)	1.03% (0.02 to 2.05)
No/unknown	88.971 (67.23)	33.087 (70.08)		91.932 (70.98)	32.238 (73.49)		19.879 (70.55)	7.493 (71.64)	
Surgery at primary site, *n* (%)									
Subtotal colectomy/hemicolectomy	110.072 (83.15)	39.223 (83.02)	0.13% (−0.25 to 0.52)	107.940 (83.40)	36.773 (83.95)	0.49% (0.1 to 0.89)	23.571 (83.75)	8.740 (83.39)	0.15% (−0.68 to 0.98)
Partial colectomy, segmental resection	19.176 (14.49)	7.136 (15.09)	0.61% (0.24 to 0.99)	18.291 (14.13)	6.197 (14.12)	0.006% (−0.37 to 0.38)	3.796 (13.46)	1.473 (14.08)	0.6% (−0.17 to 1.37)
Total colectomy	2.001 (1.51)	608 (1.29)	0.22% (0.10 to 0.34)	2.018 (1.56)	549 (1.24)	0.3% (0.18 to 0.43)	527 (1.85)	182 (1.76)	0.13% (−0.16 to 0.42)
Total proctocolectomy	365 (0.28)	102 (0.22)	0.05% (0.01 to 0.11)	376 (0.28)	114 (0.24)	0.03% (−0.02 to 0.08)	122 (0.41)	30 (0.32)	0.14% (0.02 to 0.27)
Colectomy, NOS	525 (0.40)	129 (0.27)	0.12% (0.06 to 0.18)	635 (0.48)	156 (0.34)	0.13% (0.06 to 0.2)	109 (0.37)	28 (0.30)	0.11% (−0.01 to 0.24)
Surgery, NOS	237 (0.17)	50 (0.11)	0.07% (0.04 to 0.11)	213 (0.15)	57 (0.11)	0.03% (−0.005 to 0.07)	50 (0.16)	13 (0.15)	0.05% (−0.03 to 0.13)

Abbreviations: CI, confidence interval; NCDB, National Cancer Database; NOS, not otherwise specified; SD, standard deviation; SEER, Surveillance, Epidemiology, and End Results.

^a^ Weighted‐proportional difference/standardized difference (95% CI).

**Table 5:** Tumor and treatment characteristics of primary colon cancer (left‐sided colon cancer).

Variable	Splenic flexure of colon			Descending colon			Sigmoid colon		
	NCDB *N* = 19.407	SEER *N* = 7.232	CI^a^	NCDB *N* = 37.744	SEER *N* = 13.557	CI^a^	NCDB *N* = 161.492	SEER *N* = 60.581	CI^a^
Tumor stage, *n* (%)									
Stage I	3.839 (19.77)	1.419 (19.62)	0.16% (−0.91 to 1.23)	8.893 (23.58)	3.250 (23.97)	0.41% (−0.42 to 1.24)	43.877 (27.15)	17.449 (28.77)	1.63% (1.21 to 2.05)
Stage II	7.132 (36.75)	2.654 (36.74)	0.05% (−1.24 to 1.35)	12.696 (33.62)	4.558 (33.61)	0.01% (−0.91 to 0.94)	45.351 (28.09)	16.738 (27.65)	0.45% (0.03 to 0.87)
Stage III	6.513 (33.55)	2.403 (33.20)	0.33% (−0.94 to 1.6)	12.526 (33.17)	4.390 (32.40)	0.8% (−0.11 to 1.72)	55.495 (34.37)	19.904 (32.85)	1.5% (1.06 to 1.94)
Stage IV	1.923 (9.93)	756 (10.44)	0.54% (−0.27 to 1.36)	3.629 (9.63)	1.359 (10.02)	0.4% (−0.17 to 0.99)	16.769 (10.39)	6.490 (10.73)	0.32% (0.04 to 0.61)
Grade, *n* (%)									
G1	1.875 (9.68)	576 (7.96)	1.69% (0.94 to 2.44)	3.829 (10.15)	1.183 (8.73)	1.41% (0.85 to 1.98)	16.890 (10.45)	5.572 (9.22)	1.26% (0.98 to 1.53)
G2	14.107 (72.57)	5.356 (74.05)	1.36% (0.18 to 2.55)	27.273 (72.22)	10.036 (74.03)	1.77% (0.9 to 2.63)	118.938 (73.63)	45.535 (75.08)	1.51% (1.1 to 1.92)
G3	2.627 (13.56)	994 (13.75)	0.2% (−0.72 to 1.13)	4.659 (12.36)	1.707 (12.59)	0.24% (−0.4 to 0.89)	16.597 (10.28)	6.389 (10.56)	0.26% (−0.01 to 0.55)
G4	269 (1.43)	130 (1.80)	0.41% (0.06 to 0.75)	485 (1.29)	196 (1.45)	0.16% (−0.07 to 0.39)	1.737 (1.09)	693 (1.16)	0.06% (−0.03 to 0.16)
Unknown	529 (2.76)	176 (2.44)	0.29% (−0.13 to 0.71)	1.498 (3.98)	435 (3.20)	0.76% (0.4 to 1.11)	7.330 (4.55)	2.392 (3.98)	0.59% (0.4 to 0.77)
T‐stage, *n* (%)									
T1	1.795 (9.25)	749 (10.35)	1.1% (0.29 to 1.91)	4.949 (13.13)	2.074 (15.29)	2.18% (1.49 to 2.88)	26.782 (16.58)	12.152 (20.05)	3.47% (3.1 to 3.84)
T2	2.412 (12.43)	901 (12.47)	0.03% (−0.86 to 0.92)	4.973 (13.20)	1.731 (12.75)	0.4% (−0.25 to 1.06)	24.479 (15.14)	9.189 (15.16)	0.01% (−0.32 to 0.34)
T3	11.632 (59.92)	4.554 (62.93)	3.03% (1.72 to 4.34)	21.236 (56.20)	7.884 (58.24)	1.89% (0.92 to 2.86)	82.898 (51.36)	31.664 (52.27)	0.93% (0.46 to 1.4)
T4	2.603 (13.41)	993 (13.76)	0.31% (−0.6 to 1.24)	4.770 (12.64)	1.789 (13.17)	0.16% (−0.1 to 1.21)	18.779 (11.62)	7.196 (11.88)	0.24% (−0.05 to 0.55)
Unknown	965 (4.99)	35 (0.49)	4.48% (4.14 to 4.83)	1.816 (4.83)	79 (0.55)	4.22% (3.97 to 4.47)	8.554 (5.30)	380 (0.64)	4.66% (4.54 to 4.79)
Number of retrieved regional lymph node, *n* (%)									
< 12 lymph nodes	1.495 (7.74)	672 (9.30)	1.58% (0.82 to 2.35)	2.811 (7.50)	1.309 (9.66)	2.2% (1.64 to 2.77)	13.224 (8.19)	6.163 (10.17)	1.98% (1.7 to 2.25)
12–16 lymph nodes	6.235 (32.07)	2.317 (32.03)	0.08% (−1.17 to 1.34)	11.991 (31.81)	4.254 (31.37)	0.39% (−0.52 to 1.3)	50.457 (31.23)	18.552 (30.66)	0.62% (0.18 to 1.05)
17+ lymph nodes	11.586 (59.66)	4.204 (58.12)	1.56% (0.23 to 2.89)	22.771 (60.18)	7.926 (58.50)	1.86% (0.9 to 2.83)	96.993 (60.06)	35.480 (58.54)	1.49% (1.03 to 1.95)
Unknown/missing	91 (0.53)	39 (0.55)	0.07% (−0.12 to 0.26)	171 (0.51)	68 (0.47)	0.04% (−0.08 to 0.18)	818 (0.52)	386 (0.63)	0.13% (0.05 to 0.2)
Mean (standard deviation)	18.10 (±10.89)	17.0 (±10.73)	0.1	18.38 (±11.09)	17.25 (±10.63)	0.1	17.40 (±10.17)	16.28 (±9.75)	0.1
0	11.534 (59.28)	4.297 (59.44)	0.02% (−1.31 to 1.34)	22.622 (59.89)	8.111 (59.87)	0.1% (−0.85 to 1.06)	93.111 (57.66)	35.295 (58.24)	0.6% (0.14 to 1.06)
1	2.480 (12.80)	888 (12.28)	0.5% (−0.39 to 1.39)	4.645 (12.31)	1.621 (11.95)	0.34% (−0.28 to 0.98)	20.935 (12.98)	7.449 (12.29)	0.66% (0.35 to 0.97)
2–3	2.581 (13.31)	945 (13.06)	0.23% (−0.67 to 1.14)	4.900 (12.99)	1.713 (12.63)	0.34% (−0.3 to 1.0)	21.572 (13.34)	7.800 (12.88)	0.48% (0.16 to 0.79)
4–6	1.493 (7.73)	550 (7.60)	0.08% (−0.62 to 0.8)	2.953 (7.84)	1.084 (7.98)	0.17% (−0.35 to 0.7)	13.473 (8.34)	4.944 (8.17)	0.18% (−0.07 to 0.43)
7+	609 (3.18)	224 (3.10)	0.04% (−0.42 to 0.5)	1.132 (3.01)	432 (3.18)	0.18% (−0.15 to 0.52)	5.412 (3.35)	2.000 (3.31)	0.04% (−0.11 to 0.21)
Unknown/missing	710 (3.70)	328 (4.52)	0.87% (0.32 to 1.42)	1.492 (3.96)	596 (4.39)	0.44% (0.04 to 0.84)	6.989 (4.33)	3.093 (5.11)	0.77% (0.57 to 0.97)
Mean (SD)	1.47 (±3.03)	1.46 (±3.00)	0.003	1.47 (±3.09)	1.48 (±3.12)	0.003	1.58 (±3.23)	1.54 (±3.23)	0.01
Tumor size (mm), mean (SD)	47.69 (±60.77)	44.62 (±30.72)	0.06	45.81 (±62.08)	42.30 (±25.76)	0.07	44.90 (±63.84)	41.19 (±27.42)	0.08
Tumor size (mm), median (interquartile range)	40 (25)	40 (25)		40 (28)	40 (28)		40 (26)	40 (27)	
First surgical procedure time, months from diagnosis, mean [range–day]	0.58 [0–580]	0.55 [0–690]		0.59 [0–563]	0.55 [0–720]		0.66 [0–701]	0.60 [0–630]	
Vital status, *n* (%)									
Alive	10.416 (53.53)	3.701 (51.26)	2.49% (1.14 to 3.84)	21.572 (57.02)	7.399 (54.62)	2.57% (1.6 to 3.55)	95.319 (58.98)	34.496 (56.87)	2.08% (1.62 to 2.54)
Dead	8.991 (46.47)	3.531 (48.74)		16.172 (42.98)	6.158 (45.38)		66.173 (41.02)	26.085 (43.13)	
Chemotherapy, *n* (%)									
Yes	6.987 (35.94)	2.495 (34.56)	1.5% (0.21 to 2.79)	14.180 (37.52)	4.698 (34.71)	2.91% (1.97 to 3.85)	64.208 (39.76)	21.839 (36.01)	3.7% (3.25 to 4.16)
No/unknown	12.420 (64.06)	4.737 (65.44)		23.564 (62.48)	8.859 (65.29)		97.284 (60.24)	38.742 (63.99)	
Surgery at primary site, *n* (%)									
Subtotal colectomy/hemicolectomy	10.673 (54.91)	3.777 (52.20)	2.76% (1.42 to 4.11)	23.731 (62.84)	8.225 (60.82)	2.2% (1.24 to 3.15)	32.552 (20.16)	9.460 (15.61)	4.54% (4.19 to 4.89)
Partial colectomy, segmental resection	7.923 (40.77)	3.215 (44.50)	3.62% (2.29 to 4.96)	12.050 (31.92)	4.790 (35.33)	3.4% (2.47 to 4.33)	121.026 (74.86)	49.136 (81.10)	6.16% (5.78 to 6.54)
Total colectomy	573 (2.98)	176 (2.42)	0.51% (0.09 to 0.94)	1.374 (3.65)	404 (2.94)	0.66% (0.31 to 1.0)	4.311 (2.69)	1.176 (1.94)	0.72% (0.59 to 0.86)
Total proctocolectomy	64 (0.36)	27 (0.36)	0.04% (−0.11 to 0.2)	254 (0.68)	63 (0.43)	0.21% (0.06 to 0.34)	866 (0.56)	281 (0.46)	0.07% (0.007 to 0.13)
Colectomy, NOS	107 (0.59)	33 (0.46)	0.09% (−0.09 to 0.28)	241 (0.65)	57 (0.38)	0.22% (0.08 to 0.35)	1.652 (1.04)	332 (0.56)	0.47% (0.39 to 0.55)
Surgery NOS	67 (0.39)	4 (0.06)	0.28% (0.19 to 0.38)	94 (0.26)	18 (0.10)	0.11% (0.03 to 0.19)	1.085 (0.69)	196 (0.33)	0.34% (0.28 to 0.4)

Abbreviations: CI, confidence interval; NCDB, National Cancer Database; NOS, not otherwise specified; SD, standard deviation; SEER, Surveillance, Epidemiology, and End Results.

^a^ Weighted‐proportional difference/standardized difference (95% CI).

**Table 6:** Tumor and treatment characteristics of primary colon cancer (other colon locations).

Variable	Transverse colon			Appendix		
	NCDB *N* = 59.406	SEER *N* = 21.239	CI^a^	NCDB *N* = 4.123	SEER *N* = 1.463	CI^a^
Tumor stage, *n* (%)						
Stage I	15.248 (25.66)	5.530 (26.04)	0.36% (−0.31 to 1.05)	859 (20.84)	350 (23.92)	3.08% (0.57 to 5.6)
Stage II	22.075 (37.16)	7.885 (37.16)	0.03% (−0.72 to 0.79)	1.820 (44.14)	667 (45.59)	1.44% (−1.51 to 4.41)
Stage III	17.200 (28.98)	5.958 (28.04)	0.9% (0.19 to 1.6)	872 (21.15)	270 (18.46)	2.69% (0.34 to 5.04)
Stage IV	4.883 (8.20)	1.866 (8.76)	0.56% (0.12 to 1.0)	572 (13.87)	176 (12.03)	1.84% (−0.12 to 3.81)
Grade, *n* (%)						
G1	6.086 (10.23)	1.923 (9.06)	1.19% (0.73 to 1.64)	760 (18.44)	254 (17.36)	1.07% (−1.2 to 3.34)
G2	40.516 (68.20)	14.818 (69.77)	1.56% (0.28 to 0.84)	2.320 (56.27)	856 (58.5)	2.24% (−0.7 to 5.18)
G3	9.758 (16.42)	3.485 (16.40)	0.01% (−0.56 to 0.59)	735 (17.83)	243 (16.6)	1.21% (−1.01 to 3.45)
G4	1.170 (1.98)	427 (2.01)	0.04% (−0.17 to 0.26)	29 (0.7)	11 (0.75)	0.04% (−0.46 to 0.55)
Unknown	1.876 (3.17)	586 (2.76)	0.39% (0.13 to 0.66)	279 (6.76)	99 (6.76)	0% (−1.49 to 1.49)
T‐stage, *n* (%)						
T1	7.642 (12.87)	3.102 (14.60)	1.74% (1.19 to 2.28)	355 (8.61)	162 (11.07)	2.46% (0.64 to 4.28)
T2	8.701 (14.62)	3.162 (14.88)	0.24% (−0.31 to 0.79)	578 (14.02)	217 (14.83)	0.81% (−1.29 to 2.92)
T3	33.147 (55.82)	12.323 (58.03)	2.22% (1.44 to 2.99)	1.700 (41.23)	616 (42.1)	0.87% (−2.06 to 3.81)
T4	7.109 (11.97)	2.542 (11.97)	0.002% (−0.5 to 0.51)	1.260 (30.57)	450 (30.76)	0.19% (−2.55 to 2.94)
Unknown	2.807 (4.72)	110 (0.52)	4.2% (4.01 to 4.4)	230 (5.57)	18 (1.24)	4.34% (3.44 to 5.24)
Number of retrieved regional lymph node, *n* (%)						
< 12 lymph nodes	4.227 (7.10)	1.771 (8.36)	1.22% (0.79 to 1.64)	840 (20.37)	394 (26.93)	6.55% (3.97 to 9.14)
12–16 lymph nodes	17.167 (28.89)	6.196 (29.16)	0.27% (−0.43 to 0.98)	1.041 (25.25)	360 (24.61)	0.64% (−1.93 to 3.21)
17+ lymph nodes	37.722 (63.53)	13.160 (61.94)	1.53% (0.77 to 2.29)	2.195 (53.24)	694 (47.43)	5.8% (2.82 to 8.77)
Unknown/missing	290 (0.48)	112 (0.54)	0.04% (−0.07 to 0.15)	47 (1.14)	15 (1.03)	0.11% (−0.49 to 0.72)
Mean (standard deviation)	19.31 (±12.08)	18.23 (±11.6)	0.09	15.52 (±11.34)	13.82 (±11.48)	0.14
Number of positive regional lymph node, *n* (%)						
0	38566 (64.90)	13768 (64.81)	0.09% (−0.65 to 0.84)	2.390 (57.96)	809 (55.3)	2.67% (−0.28 to 5.63)
1	6962 (11.71)	2463 (11.60)	0.12% (−0.37 to 0.62)	362 (8.78)	120 (8.2)	0.57% (−1.07 to 2.22)
2–3	6575 (11.07)	2328 (10.96)	0.1% (−0.38 to 0.59)	347 (8.42)	112 (7.66)	0.76% (−0.84 to 2.36)
4–6	3736 (6.29)	1326 (6.25)	0.04% (−0.33 to 0.42)	216 (5.24)	58 (3.96)	1.27% (0.06 to 2.48)
7+	1488 (2.50)	509 (2.40)	0.1% (−0.13 to 0.34)	87 (2.11)	21 (1.44)	0.67% (−0.07 to 1.42)
Unknown/missing	2079 (3.49)	845 (3.98)	0.47% (0.17 to 0.78)	721 (17.49)	343 (23.44)	5.95% (3.49 to 8.41)
Mean (SD)	1.25 (±2.94)	1.23 (±2.83)	0.006	1.25 (±3.02)	1.06 (±2.78)	0.07
Tumor size (mm), mean (SD)	46.0 (±60.73)	43.33 (±32.98)	0.05	36.62 (±59.45)	32.14 (±22.82)	0.1
Tumor size (mm), median (interquartile range)	40 (30)	40 (30)		29 (30)	26 (30)	
First surgical procedure time, months from diagnosis, mean [range–day]	0.62 [0–598]	0.59 [0–570]		0.20 [0–380]	0.14 [0–520]	
Vital status, *n* (%)						
Alive	31.260 (52.62)	10.700 (50.38)	2.24% (1.45 to 3.02)	2.374 (57.58)	834 (57.0)	0.57% (−2.37 to 3.52)
Dead	28.146 (47.38)	10.539 (49.62)		1.749 (42.42)	629 (43.0)	
Chemotherapy, *n* (%)						
Yes	17.467 (29.4)	5.924 (27.89)	1.51% (0.8 to 2.21)	1.658 (40.21)	529 (36.16)	4.05% (1.17 to 6.93)
No/unknown	41.939 (70.6)	15.315 (72.11)		2.465 (59.79)	934 (63.84)	
Surgery at primary site, *n* (%)						
Subtotal colectomy/hemicolectomy	33.914 (57.09)	11.619 (54.7)	2.38% (1.6 to 3.16)	2.718 (65.92)	902 (61.66)	4.26% (1.38 to 7.14)
Partial colectomy, segmental resection	22.553 (37.96)	8.799 (41.43)	3.46% (2.69 to 4.23)	1.081 (26.22)	491 (33.56)	7.34% (4.57 to 10.1)
Total colectomy	1.935 (3.26)	593 (2.79)	0.46% (0.2 to 0.72)	50 (1.21)	13 (0.88)	0.32% (−0.26 to 0.9)
Total proctocolectomy	351 (0.59)	95 (0.45)	0.14% (0.03 to 0.25)	14 (0.34)	7 (0.48)	0.13% (−0.25 to 0.53)
Colectomy, NOS	498 (0.84)	106 (0.5)	0.33% (0.21 to 0.45)	14 (0.34)	2 (0.14)	0.2% (−0.05 to 0.46)
Surgery NOS	155 (0.26)	27 (0.13)	0.13% (0.07 to 0.19)	246 (5.97)	48 (3.28)	2.68% (1.52 to 3.85)

Abbreviations: CI, confidence interval; NCDB, National Cancer Database; NOS, not otherwise specified; SD, standard deviation; SEER, Surveillance, Epidemiology, and End Results.

^a^ Weighted‐proportional difference/standardized difference (95% CI).

**Figure 3:**
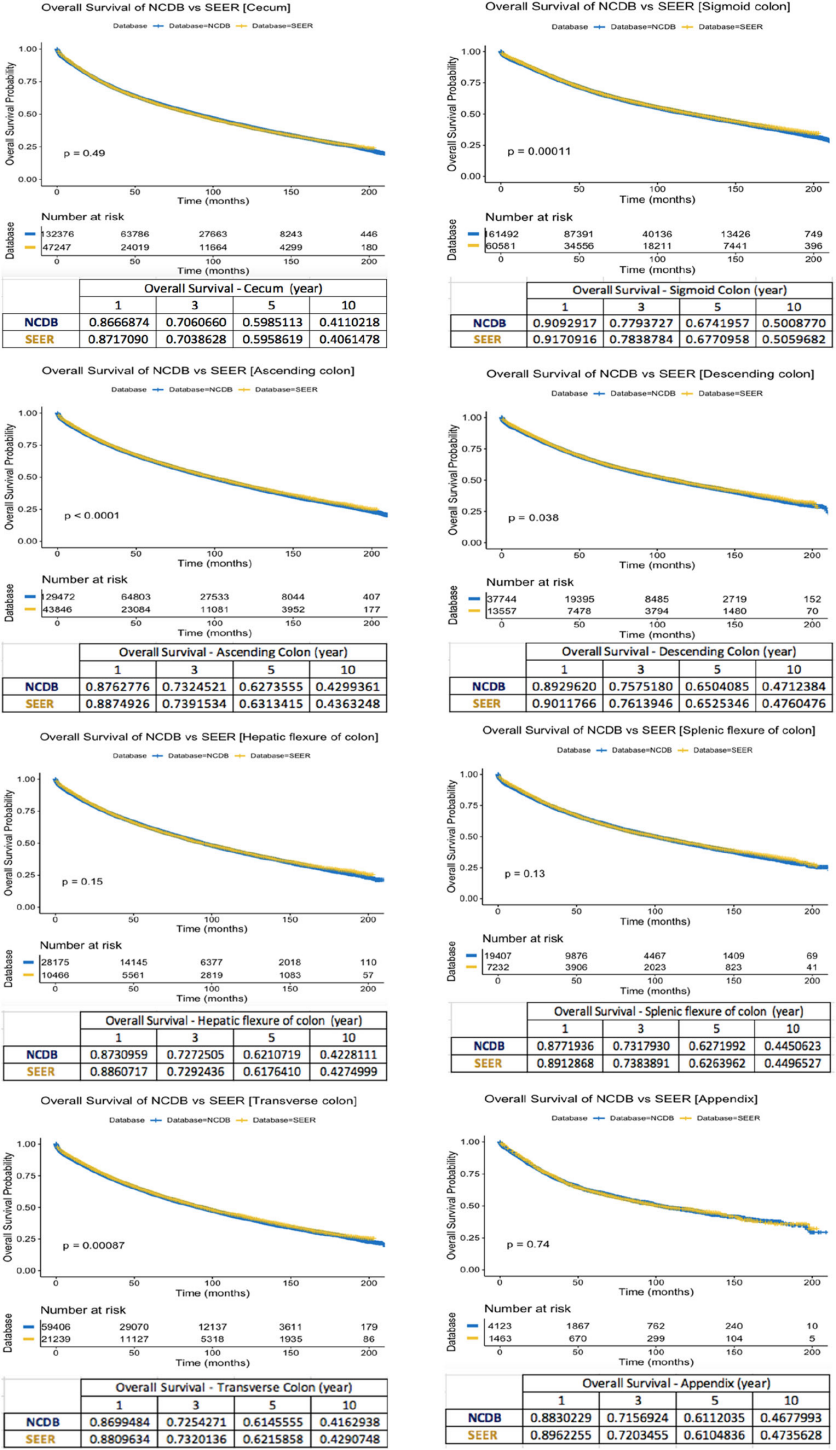
Overall survival rates of NCDB and SEER database.

KM survival estimates for OS across different colon cancer locations in NCDB and SEER are presented in Figure 3. While survival trends remain largely consistent between databases, some differences were observed in 5‐year OS rates for specific tumor locations. In the ascending colon, transverse colon, descending colon, and sigmoid colon, the 5‐year OS rates were slightly lower for NCDB cases (62.7%, 61.4%, 65%, and 67.4%, respectively) compared to SEER data (63.1%, 62.1%, 65.2%, and 67.7%, respectively). Conversely, in the cecum, appendix, hepatic flexure, and splenic flexure, NCDB reported slightly higher 5‐year OS rates (41.1%, 61.1%, 62.1%, and 62.7%, respectively) compared to SEER (40.6%, 61%, 61.7%, and 62.6%, respectively). Stage‐specific OS trends remained consistent between NCDB and SEER across most tumor locations (Supporting Information S2: Figure S1, Supporting Information S3: Figure S2, Supporting Information S4: Figure S3, Supporting Information S5: Figure S4).

Regarding differences in OS across colon cancer locations, both NCDB and SEER demonstrated similar patterns, with distinct survival trends based on tumor site. Pronounced survival distinctions were particularly evident in Stage III disease. In unadjusted analyses, the descending colon exhibited better survival than the ascending colon, with NCDB showing 3.26%, 1.35%, and 6.3% higher OS rates in Stages I–III, respectively, while SEER reported 3.22%, 1.6%, and 6.53% higher OS rates in the same stages. Similarly, in unadjusted comparisons, the splenic flexure demonstrated superior survival compared to the hepatic flexure, with NCDB reporting OS advantages of 2.0%, 0.59%, and 5.52% in Stages I–III, respectively, while SEER showed corresponding differences of 1.38%, 1.28%, and 5.38%.

## 4 Discussion

In our study, we found a consistent pattern in OS rates between NCDB and SEER across various colon cancer locations and stages, with only minor differences observed at 1‐, 3‐, 5‐, and 10‐year follow‐up points. Despite differences in sampling methodologies and data collection, both databases exhibited substantial similarity in demographic, oncologic, and treatment data. However, these similarities do not eliminate the impact of database‐specific methodologies, including differences in patient selection, variable definitions, and follow‐up reporting, all of which influence how survival outcomes are captured and interpreted. Understanding these methodological differences is critical for researchers when choosing a database for colon cancer survival analysis.

Fundamentally, assessing colon cancer disparities requires population‐based data, making NCDB and SEER two of the largest and most widely used cancer registries in the United States. However, these databases differ significantly in sampling methodology, population coverage, and data reporting, all of which influence research outcomes. NCDB is a hospital‐based registry, capturing cases from CoC‐accredited facilities, while SEER is a population‐based registry, covering a geographically diverse subset of the U.S. population.

One key difference is racial and geographic representation. NCDB contains a slightly higher percentage of Caucasian patients across all colon cancer locations, whereas SEER reports a notable geographic variance, particularly with a higher proportion of cases from the Pacific region. Given that a higher percentage of the Asian population in the United States resides in Pacific regional states such as California and Hawaii, this geographic distribution likely contributes to the increased number of Asian cases recorded in SEER compared to NCDB, aligning with findings from previous research studies [1, 3]. The differences in the number of retrieved regional lymph nodes between NCDB and SEER across colonic locations may reflect variations in surgical approaches and staging practices within databases.

Despite these methodological differences, our study demonstrates that NCDB and SEER are relatively similar in terms of patient demographics, oncologic data, and treatment variables. Similarly, Mettlin et al. [1] found no significant differences in age and gender between the NCDB and SEER in their analysis comparing breast, colorectal, lung, and prostate cancers. In addition to that, Janz et al. [2] and Yolcu et al. [3] found no discrepancies between the NCDB and SEER database in terms of demographics, oncologic, and treatment variables in head and neck cancers and primary central nervous tumors, respectively. However, our study expands upon previous research in several key ways. First, we conducted a more comprehensive analysis specifically in colon cancer, a disease with high variability in tumor location and stage‐dependent treatment approaches, whereas prior studies focused on other cancer types. By incorporating a broader set of variables and a larger dataset, we provided a more detailed comparison of database differences. Second, unlike previous studies that primarily examined overall database characteristics, we stratified and analyzed OS rates across different colon cancer locations, offering a more comprehensive evaluation of potential methodological biases and variations in perioperative factors. While NCDB primarily captures patients treated at CoC‐accredited hospitals and SEER represents a broader population‐based cohort, survival differences may reflect variations in treatment access, institutional referral patterns, and data reporting methodologies rather than true differences in clinical outcomes. Previous systematic reviews and randomized clinical trials have provided inconclusive evidence regarding the survival benefits of intensive surveillance programs compared to less rigorous follow‐up strategies [13, 14, 15, 16]. In our study, although we observed statistically significant differences in OS rates between NCDB and SEER for certain colon cancer locations based on KM estimates, these differences were relatively minor and may not translate into clinically meaningful disparities. Moreover, despite these structural differences, OS trends between the databases were similar. Our findings align with the literature suggesting that tumor characteristics may have a greater influence on OS than follow‐up status or treatment setting [13, 17].

In our study, notable differences in 5‐year OS rates were observed across colonic regions, particularly in Stage III disease. The survival advantage of the descending colon over the ascending colon and the splenic flexure over the hepatic flexure became more pronounced at Stage III, while differences in earlier stages were smaller. A similar trend is observed between the sigmoid colon and cecum, with Stage III survival favoring the sigmoid colon. In the literature, studies on colon cancer survival have primarily focused on comparisons between right‐sided and left‐sided colon cancers. Turner et al. [4] and Warschkow et al. [8] reported that Stages I and II left‐sided colon cancer demonstrated lower survival rates compared to right‐sided colon cancer, while patients with left‐sided stage III disease demonstrated a survival advantage by using the NCDB and the SEER databas, respectively. However, certain studies found either no significant difference in terms of 5‐year OS rates between right and left‐sided colon cancers [18] or indicated lower 5‐year OS rates in right‐sided colon cancers. Weiss et al. [7] and Meguid et al. [6] stated that right‐sided colon cancers exhibit lower 5‐year OS rates across nearly all stages, except for Stage II, whereas some studies have shown consistently lower 5‐year OS rates in right‐sided colon cancers [19, 20, 21, 22]. The comparison of 5‐year OS rates between the right and left‐sided colon cancers was also conducted using specific hospital databases. Wang et al. [23] found no significant differences in 5‐year OS rates for Stages I–III between right and left‐sided colon cancers. However, in the case of Stage IV, left‐sided cancers show significantly more survival advantage. Given these points, the existing literature presents conflicting findings regarding the prognosis of right‐sided and left‐sided colon cancers, with variations in reported survival rates across different stages. Researchers have obtained different results based on their selected methodologies when comparing right‐sided colon cancer to left‐sided colon cancer, particularly in determining which side the difference ratio favors between the locations in Stages I–III.

Given these conflicting results, differences in reported OS rates are likely driven by study methodologies rather than true biological disparities. Variability arises from how colon cancer locations are categorized, the use of risk adjustment and methods to eliminate potential confounders, and whether chemotherapy administration is accounted for. Differences in inclusion and exclusion criteria further contribute to discrepancies. Moreover, inconsistent definitions of right‐ and left‐sided tumors across studies further complicate comparisons. Additionally, the variation in OS magnitudes between specific tumor locations (e.g., hepatic flexure, ascending colon, cecum vs. splenic flexure, descending colon, sigmoid colon) may persist even after statistical adjustments. While matched analyses and risk‐adjusted models may alter survival differences in Stages I and II, the greater survival advantage observed in Stage III left‐sided tumors remains prominent, suggesting that methodological inconsistencies or incomplete adjustments in certain studies may contribute to discrepancies in the literature when comparing OS outcomes in NCDB and SEER.

These inconsistencies highlight the challenges of conducting large‐scale medical research using extensive datasets such as NCDB and SEER. Large datasets introduce methodological hurdles, including data quality issues, inconsistencies in reporting, and database stability concerns. The process of data cleaning and standardization is crucial to ensuring valid conclusions, as even minor inconsistencies in database variables can produce statistically significant but misleading results [24, 25]. Given these complexities, our findings suggest that differences in survival outcomes between NCDB and SEER are likely driven by database‐specific methodologies rather than true biological variation.

Our study has several limitations that should be considered when interpreting the results. One key limitation is the difference in follow‐up reporting between NCDB and SEER. SEER determines survival status through state registries and national death certificates, ensuring more comprehensive long‐term survival tracking. In contrast, NCDB relies on hospital‐reported follow‐up data, which may result in underreporting of long‐term survival, particularly for patients who receive follow‐up care outside of CoC‐accredited hospitals. These follow‐up discrepancies could influence survival estimates and partially explain differences in OS rates between the databases. Changes in database structures over time may introduce heterogeneity in the data. SEER's expanding regional coverage led to a broader and more diverse patient population, while NCDB's hospital‐based participation fluctuated as institutions joined or left the registry. These shifts should be considered when interpreting longitudinal survival trends. Another consideration is the potential overlap of patient records between NCDB and SEER in certain regions. Since some hospitals contribute data to both databases, it is possible that some patients are registered in both datasets. However, due to patient ID blinding, the extent of this overlap is unknown, making it difficult to assess its impact on survival comparisons. Additionally, our study focused on patients who underwent surgery, which may have led to the exclusion of some stage IV patients who did not undergo resection. This decision was made to ensure the accuracy of staging data and improve comparability between NCDB and SEER. However, it limits the generalizability of our findings to stage IV patients who were managed non‐surgically. Finally, while our study aimed to compare patient characteristics, oncologic outcomes, and OS rates across anatomical locations and cancer stages between these two major databases, we did not assess OS differences among different anatomical colon locations in relation to various treatment modalities. Such an analysis would require extensive adjustments for confounders, which were beyond the scope of this study. Future research should focus on integrating multiple datasets, adjusting for key confounders, and utilizing alternative survival metrics, such as cancer‐specific survival, to improve the accuracy of database‐driven survival comparisons.

Despite these limitations, the NCDB and SEER databases provide crucial insights into cancer care in the United States, significantly contributing to the enhancement of healthcare quality. Future research should focus on adjusting for key confounders and comparing findings with similar methodologically rigorous studies to contextualize results within the broader literature. To maximize the utility of these databases and effectively address their limitations, researchers and database users should be aware of both the strengths and weaknesses of NCDB and SEER when interpreting survival outcomes and designing studies.

## 5 Conclusion

The NCDB and SEER databases provide valuable insights into colon cancer outcomes. Despite differences in sampling methodologies and follow‐up reporting, survival trends were largely consistent, with only minor variations across the databases. A clear understanding of NCDB and SEER methodologies is essential for accurate survival analysis, minimizing bias, and improving the reliability of population‐based cancer research.

## 6 Future Directions

Database selection should be guided by the specific research question to ensure the most appropriate dataset is used. Future studies may focus on integrating multiple datasets, enabling researchers to leverage the strengths of both SEER and NCDB for more comprehensive analyses. SEER contains extensive data on regional factors, including county‐level information on poverty, unemployment, and migration, as well as socioeconomic indicators at the census tract level. It is the primary database for age‐adjusted incidence and mortality rates, along with cause‐specific survival, making it essential for studying racial, ethnic, and socioeconomic disparities in cancer trends and outcomes. On the other hand, NCDB provides detailed information on tumor characteristics and treatment aspects not available in SEER, such as microsatellite instability, KRAS mutations, palliative care details, unplanned hospital readmissions, and 30‐day mortality rates following surgery. These features make NCDB particularly useful for assessing treatment efficacy and patient outcomes. For instance, when examining disparities in colon cancer related to demographics, race, ethnicity, and socioeconomic status, SEER may be the more suitable option, even though NCDB includes similar variables. This is because SEER provides more comprehensive data in these areas. Conversely, when analyzing factors influencing perioperative mortality, genetic mutations, or treatment approaches in colon cancer, NCDB may be a more appropriate database due to its detailed clinical and hospital‐based treatment data.

## Conflicts of Interest

Emre Gorgun receives consultancy fees from Boston Scientific, and DiLumen. The other authors declare no conflicts of interest.

## Synopsis

This study provides a comprehensive methodological and survival comparison of two major United States cancer databases, the National Cancer Database (NCDB) and the Surveillance, Epidemiology, and End Results (SEER) database, in the context of colon cancer research. By analyzing over 770 000 patients who underwent surgical resection for Stages I–IV colon cancer, the study examines how differences in database structure, patient representation, and follow‐up reporting influence overall survival outcomes. Although the two databases differ in data collection methods and demographic profiles, they demonstrate broadly consistent survival trends across tumor locations and disease stages. The study highlights the importance of understanding database‐specific attributes, such as the detailed treatment information captured by the hospital‐based NCDB and the broader population coverage provided by SEER. These findings serve as a methodological guide for researchers selecting the most appropriate database for colon cancer studies, whether focused on treatment patterns, disparities, or long‐term survival.

## Supporting information

STROBE Statement.

Supporting Figure 1: Stage I Overall Survival Rates of NCDB and SEER Database.

Supporting Figure 2: Stage II Overall Survival Rates of NCDB and SEER Database.

Supporting Figure 3: Stage III Overall Survival Rates of NCDB and SEER Database.

Supporting Figure 4: Stage IV Overall Survival Rates of NCDB and SEER Database.

Supporting Figure 5: Comparison of Area‐Based Rurality Measures Across NCDB and SEER Databases.

## Data Availability

The data used in this study were obtained from the National Cancer Database (NCDB) and the Surveillance, Epidemiology, and End Results (SEER) Program. Access to the NCDB was granted through an application process coordinated by the American College of Surgeons and the American Cancer Society. Access to SEER data was granted via SEER*Stat software provided by the National Cancer Institute. Due to data use agreements, the datasets are not publicly available but may be obtained through formal request to the respective organizations. Restrictions apply to the availability of these data, which were used under license for this study. Data are available from https://www.facs.org/quality-programs/cancer-programs/national-cancer-database/pufhttps://seer.cancer.gov/data/ with the permission of NCDB and SEER. The NCDB is a joint project of the Commission on Cancer of the American College of Surgeons and the American Cancer Society. The data used in this study are derived from a de‐identified NCDB file. The American College of Surgeons and the Commission on Cancer have not verified and are not responsible for the analytic or statistical methodology employed, or the conclusions drawn from these data by the investigators.
