# Supplementary figures and images for: A Methodological and Survival Comparison of NCDB and SEER Database for Colon Cancer Research
Source: J Surg Oncol. 2025 May 30;132(1):114–34. doi: 10.1002/jso.28141 (PMC12311401; doi:10.1002/jso.28141)

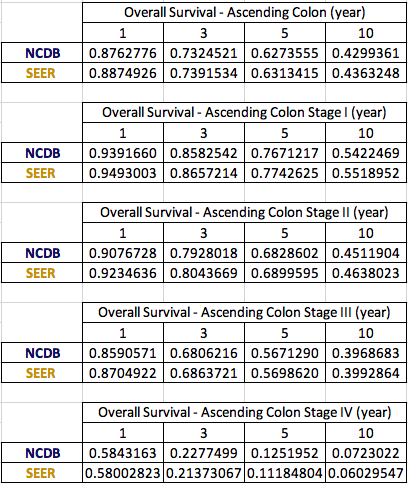

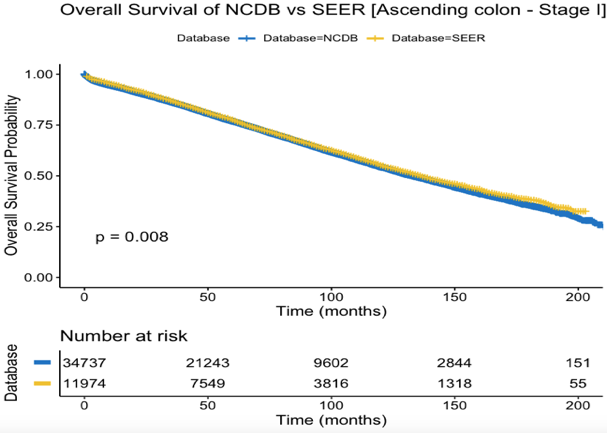

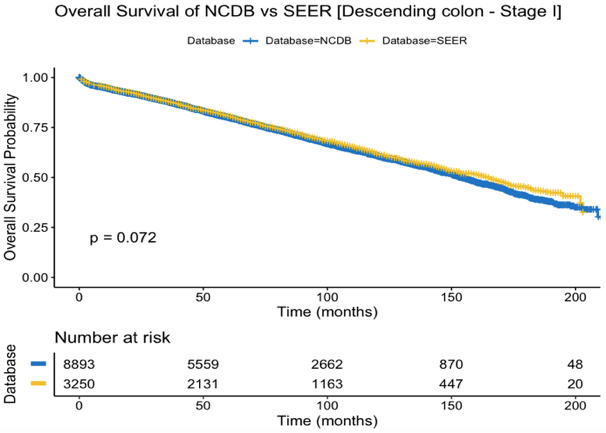

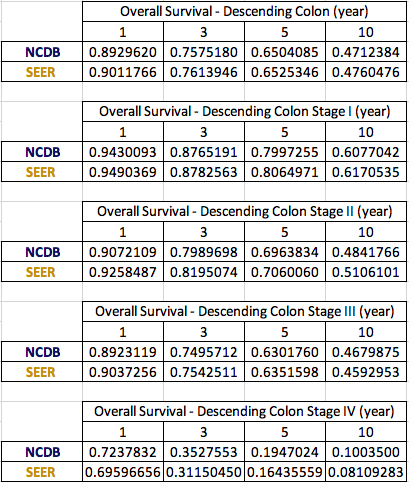

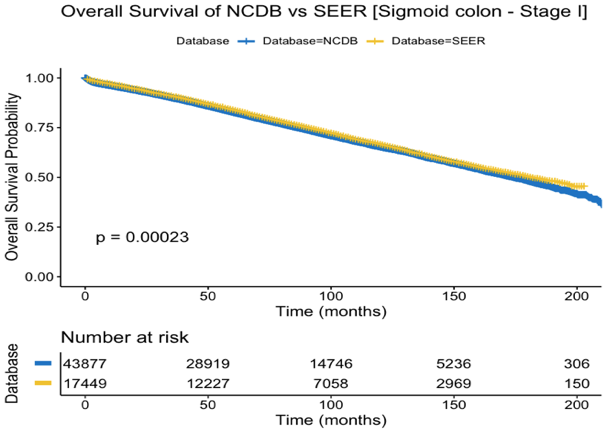

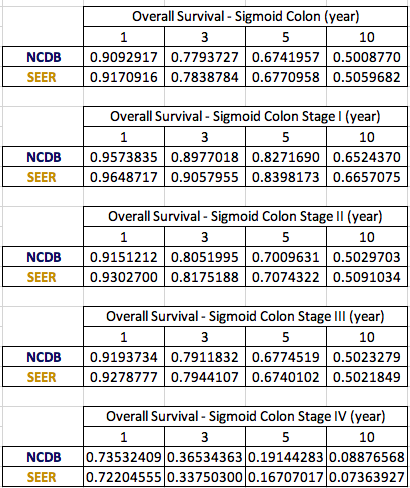

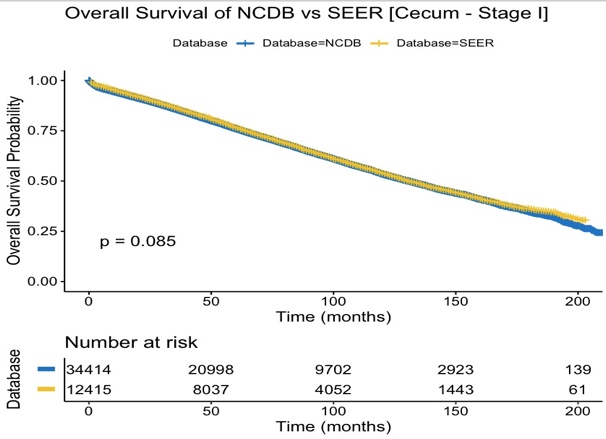

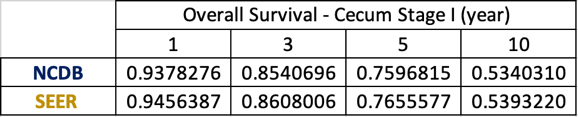

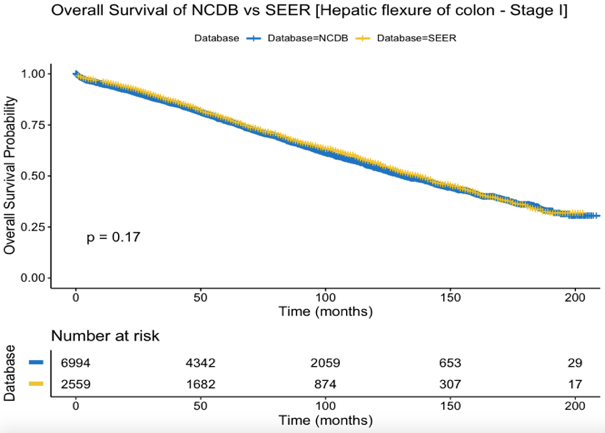

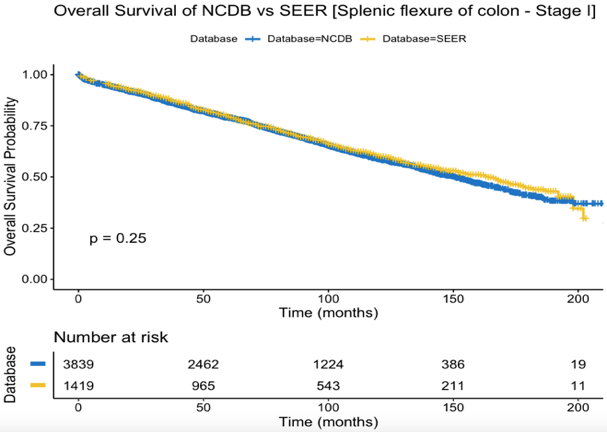

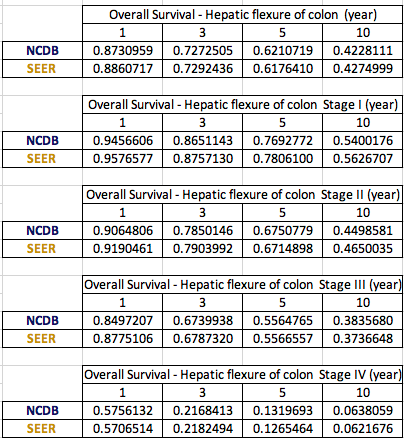

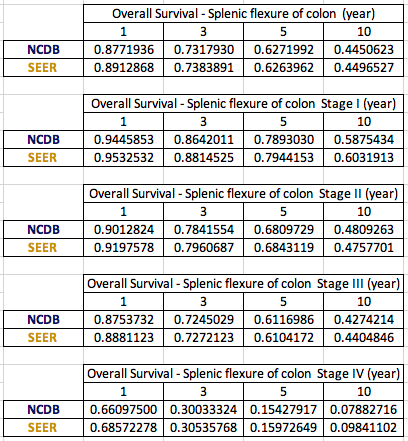

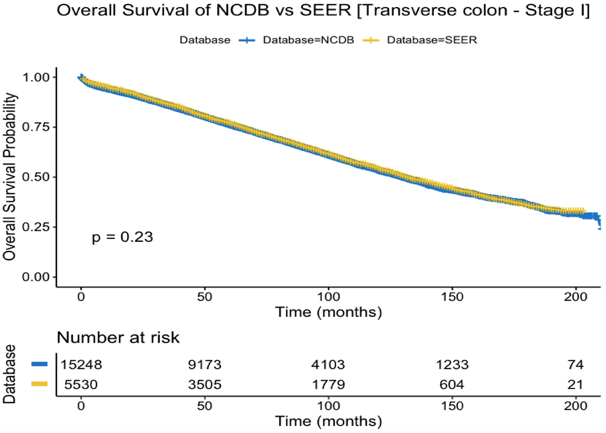

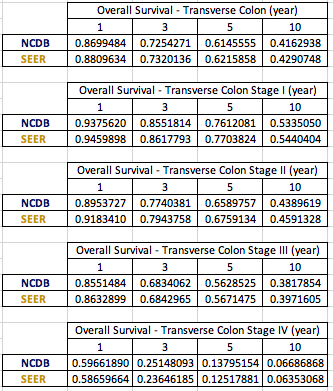

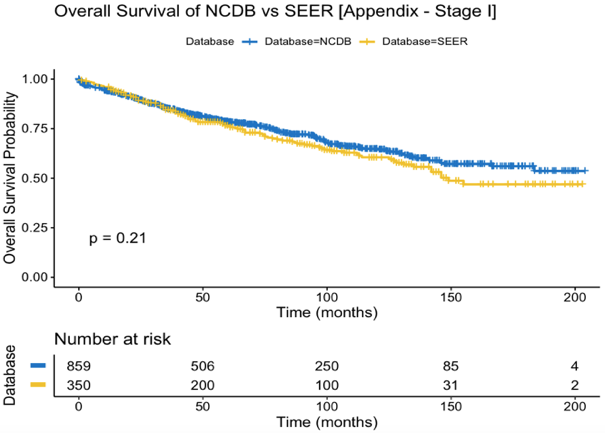

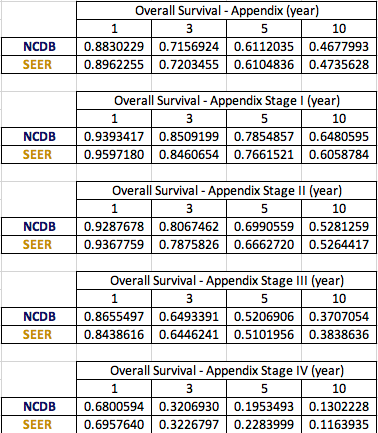


Supplemental Figure 1 Stage I Overall Survival Rates of NCDB and SEER Database

Supplement: Supplementary file 2 — Supporting Figure 1: Stage I Overall Survival Rates of NCDB and SEER Database. [file JSO-132-114-s005.docx]

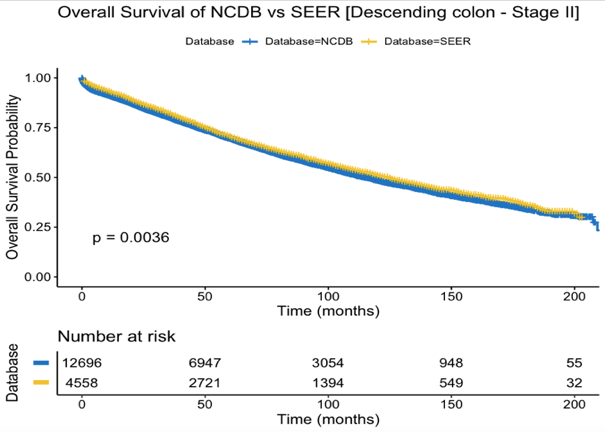

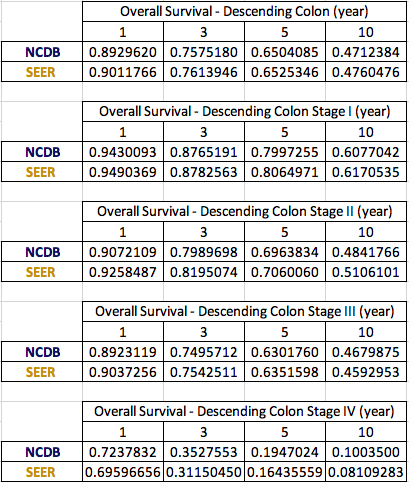

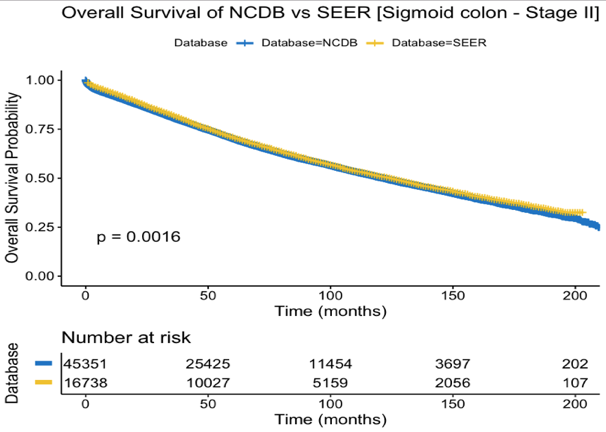

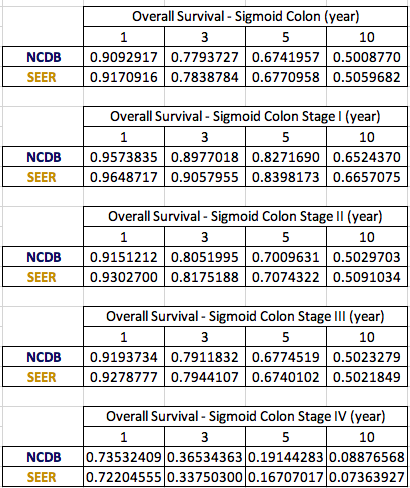

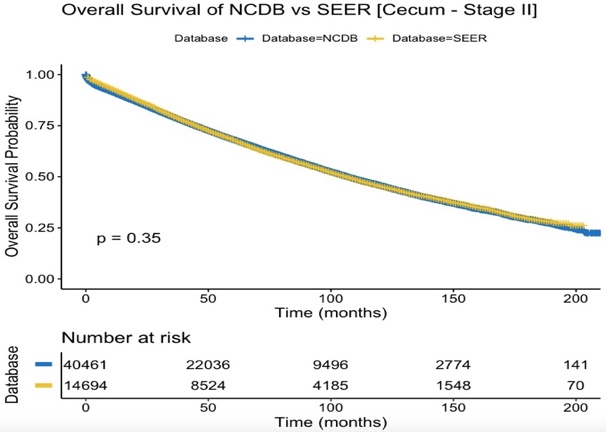

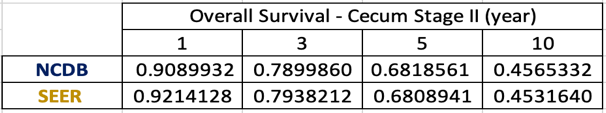

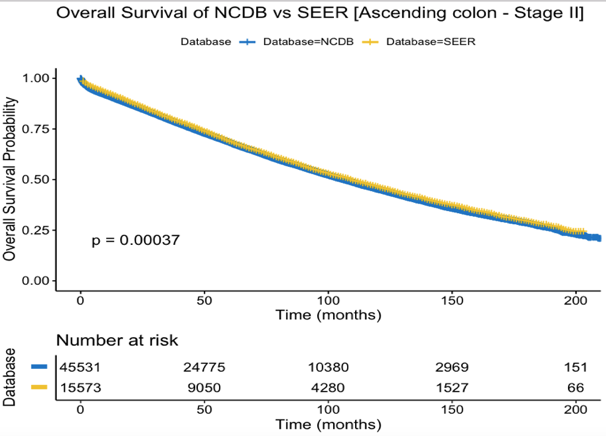

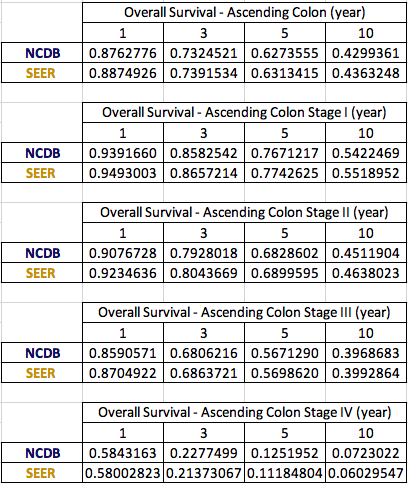

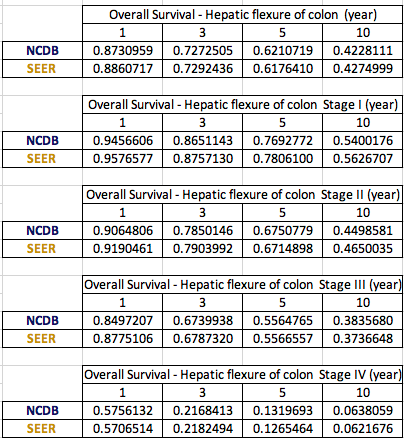

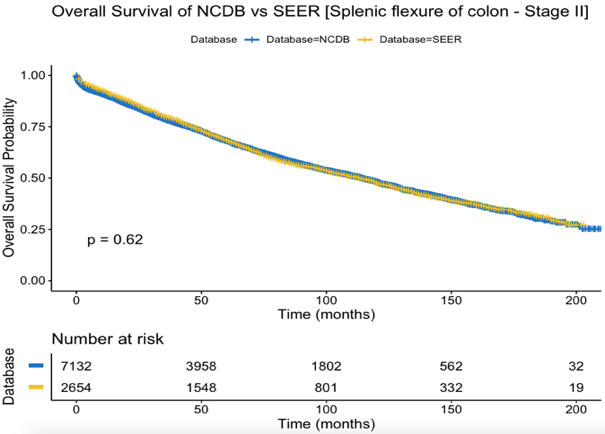

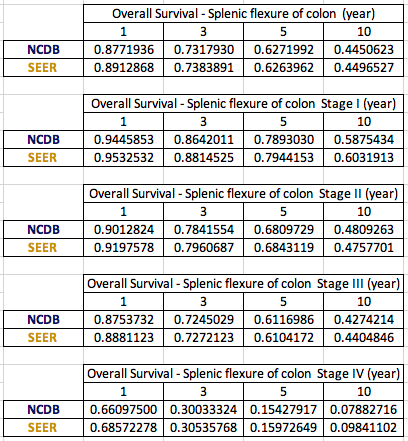

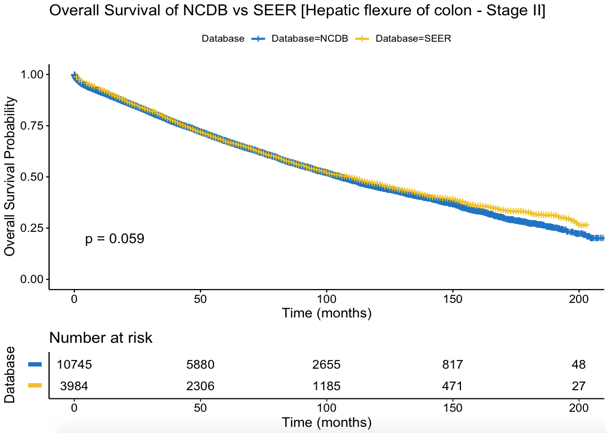

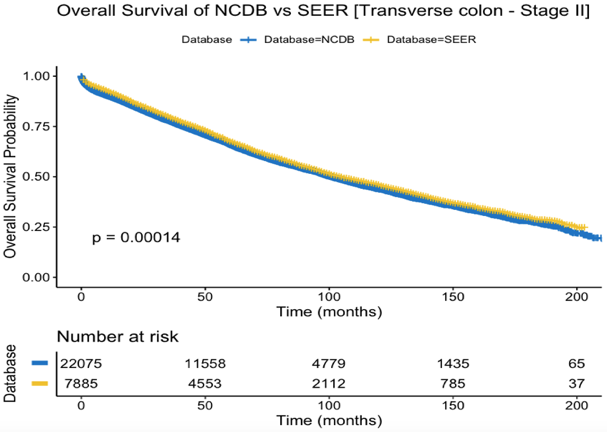

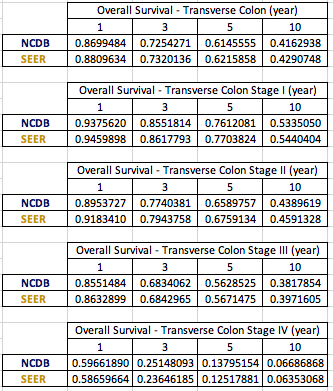

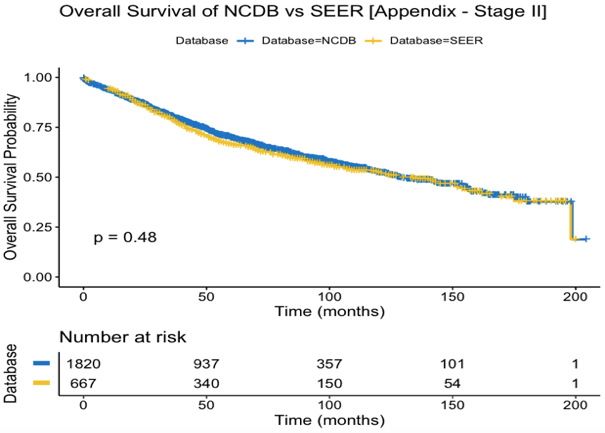

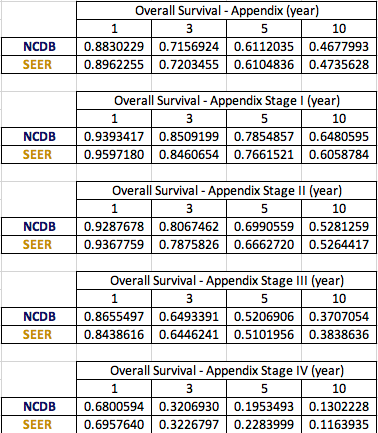


Supplemental Figure 2 Stage II Overall Survival Rates of NCDB and SEER Database

Supplement: Supplementary file 3 — Supporting Figure 2: Stage II Overall Survival Rates of NCDB and SEER Database. [file JSO-132-114-s002.docx]

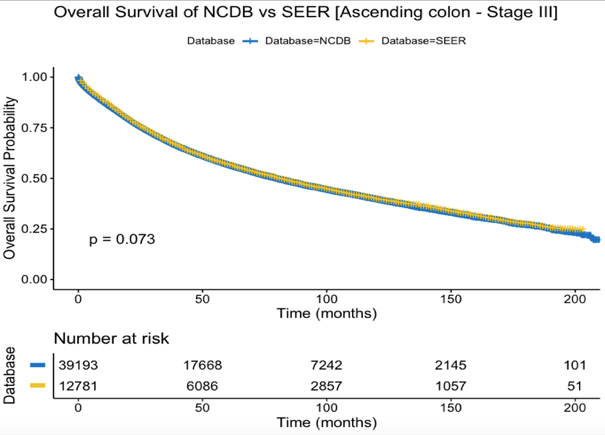

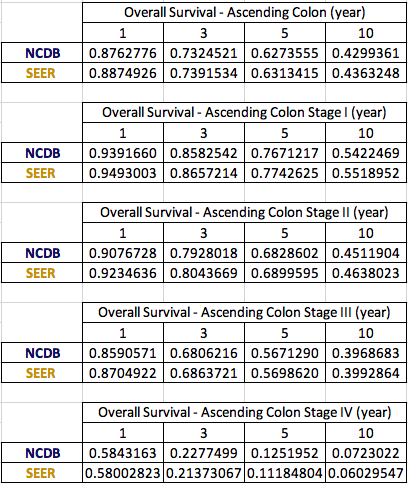

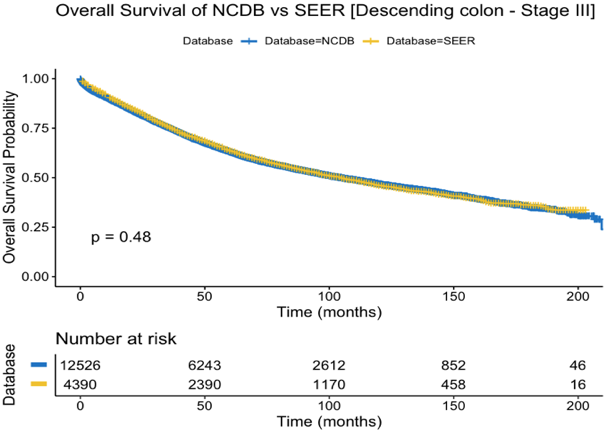

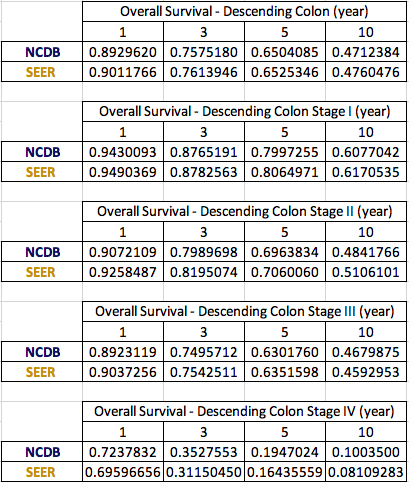

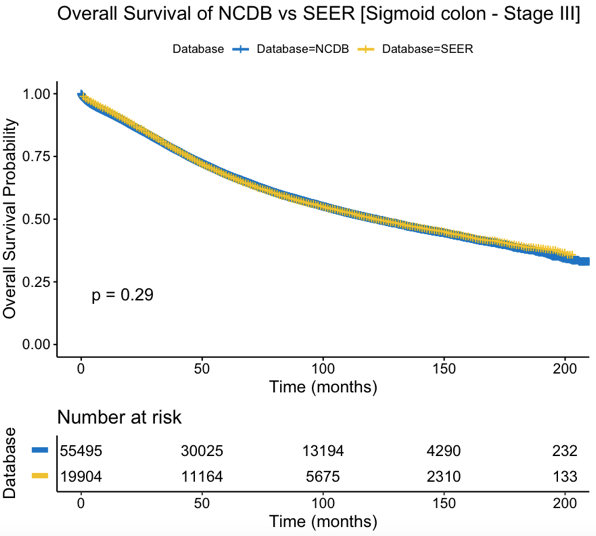

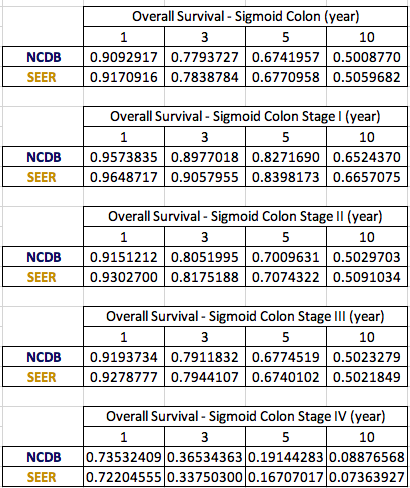

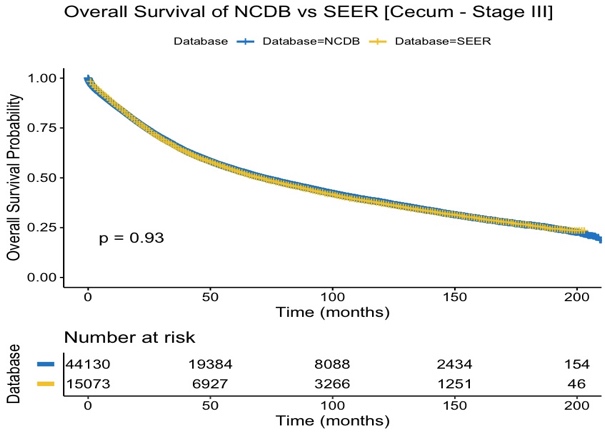

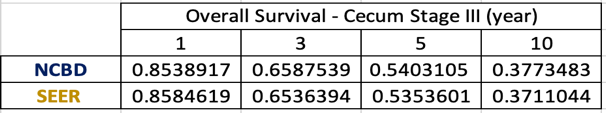

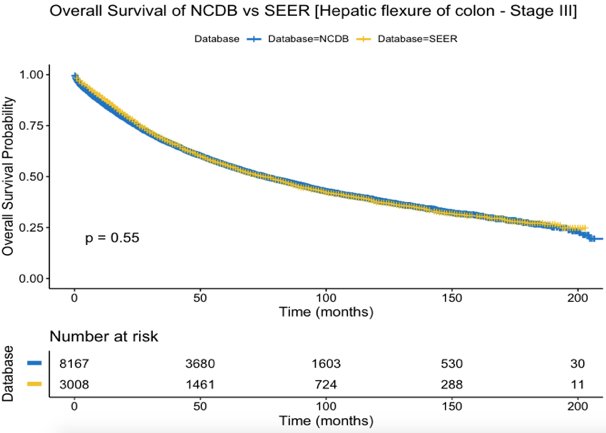

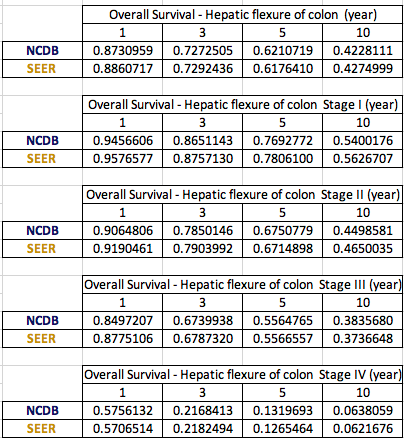

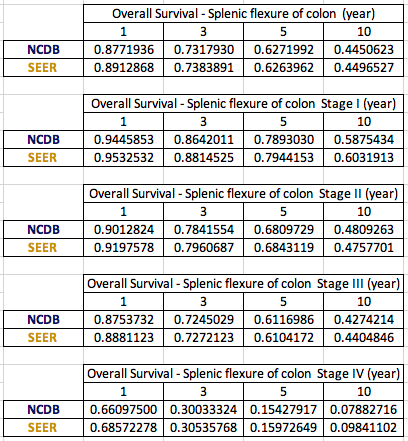

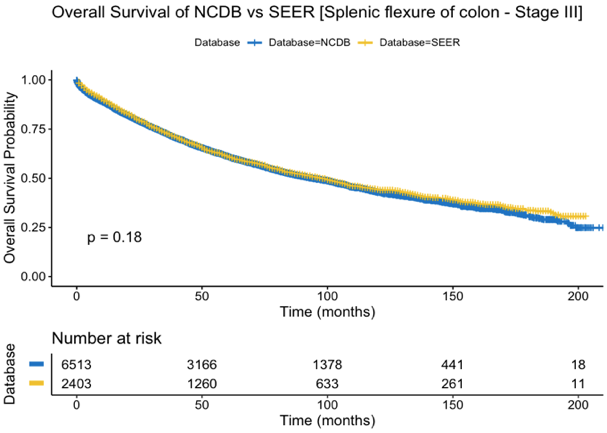

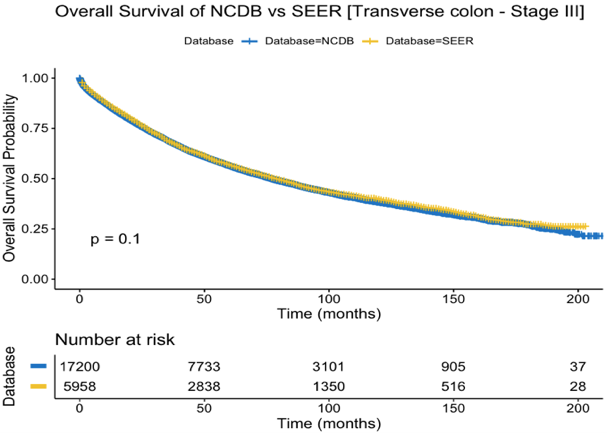

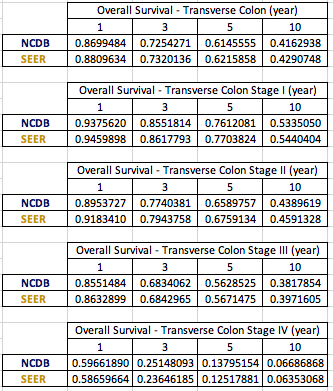

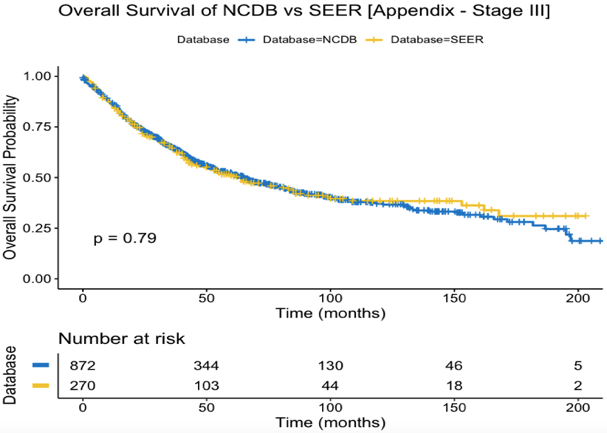

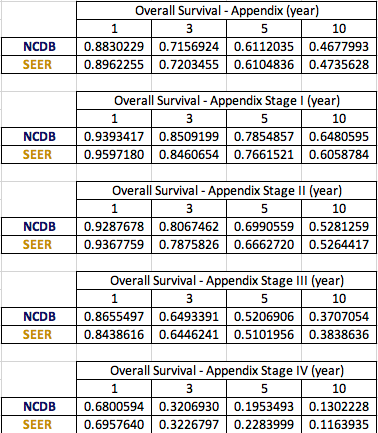


Supplemental Figure 3 Stage III Overall Survival Rates of NCDB and SEER Database

Supplement: Supplementary file 4 — Supporting Figure 3: Stage III Overall Survival Rates of NCDB and SEER Database. [file JSO-132-114-s004.docx]

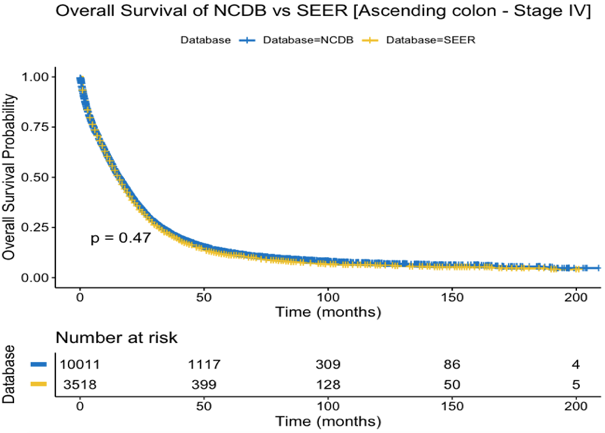

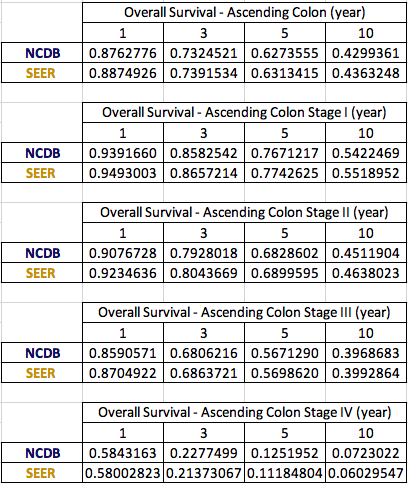

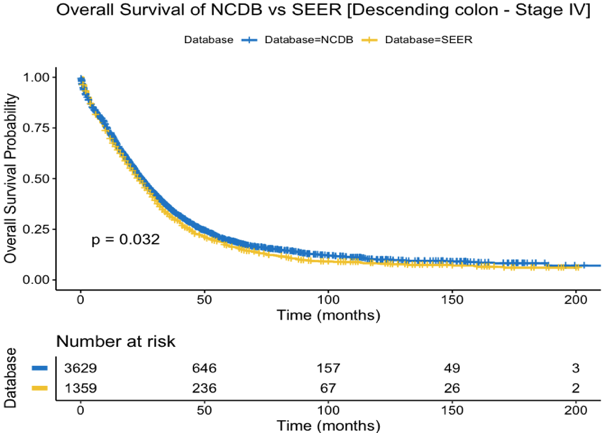

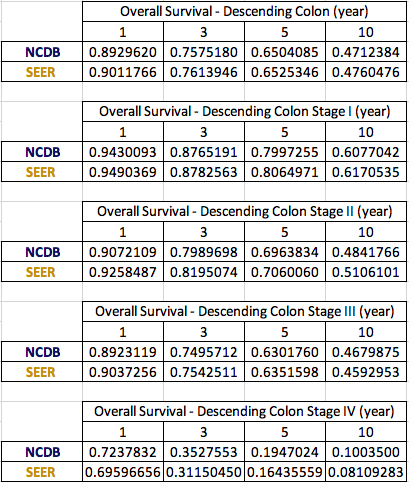

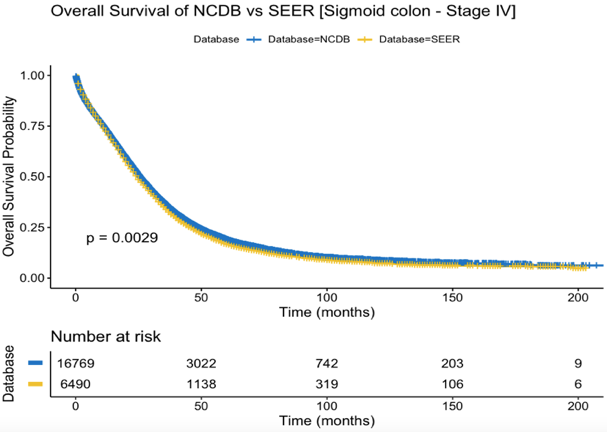

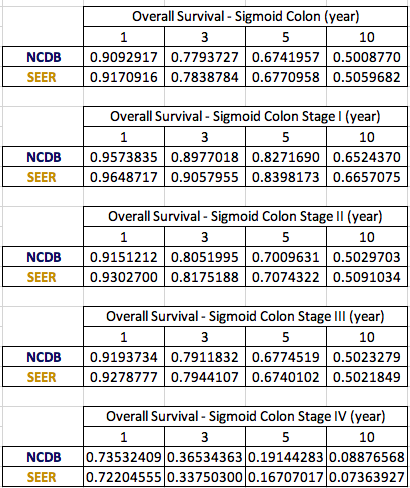

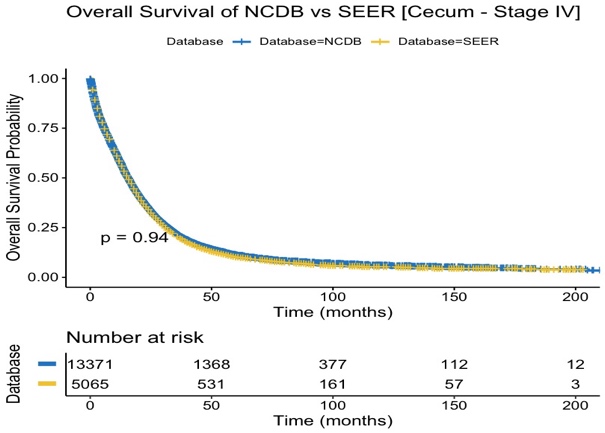

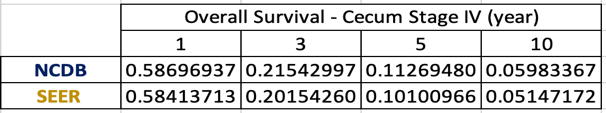

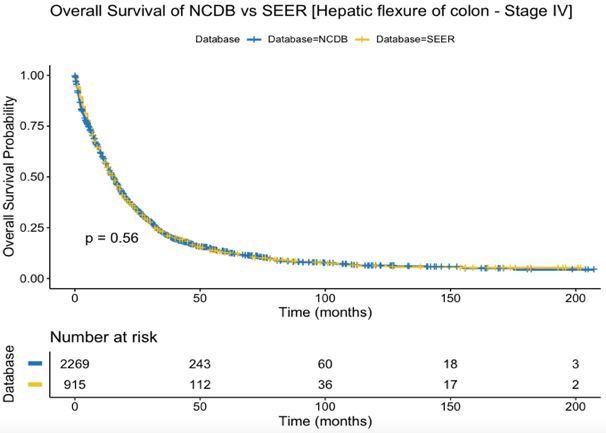

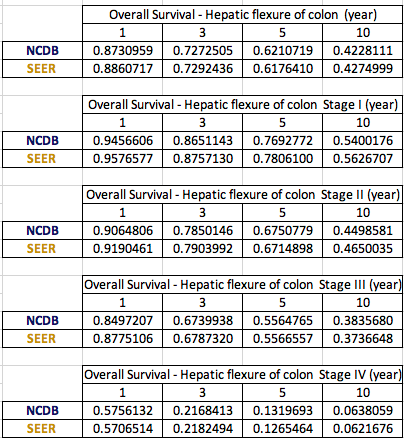

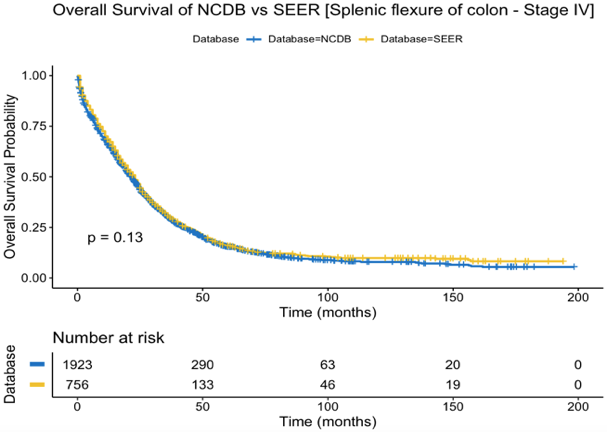

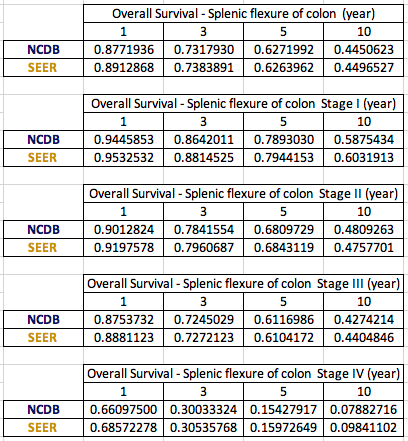

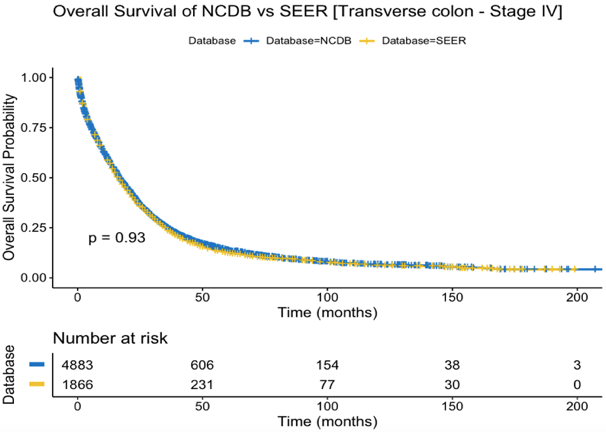

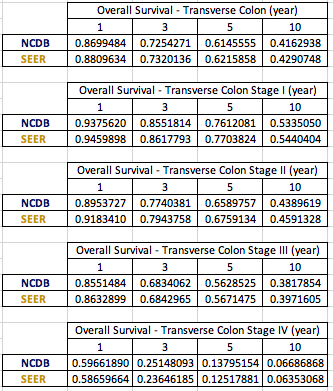

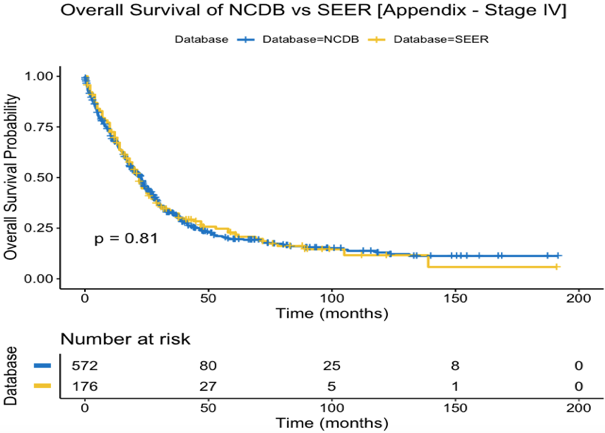

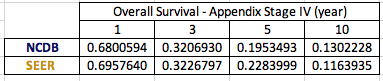


Supplemental Figure 4 Stage IV Overall Survival Rates of NCDB and SEER Database

Supplement: Supplementary file 5 — Supporting Figure 4: Stage IV Overall Survival Rates of NCDB and SEER Database. [file JSO-132-114-s003.docx]
