# Supplementary material for: A Methodological and Survival Comparison of NCDB and SEER Database for Colon Cancer Research
Source: J Surg Oncol. 2025 May 30;132(1):114–34. doi: 10.1002/jso.28141 (PMC12311401; doi:10.1002/jso.28141)
Supplement: Supplementary file 6 — Supporting Figure 5: Comparison of Area‐Based Rurality Measures Across NCDB and SEER Databases. [file JSO-132-114-s001.docx]

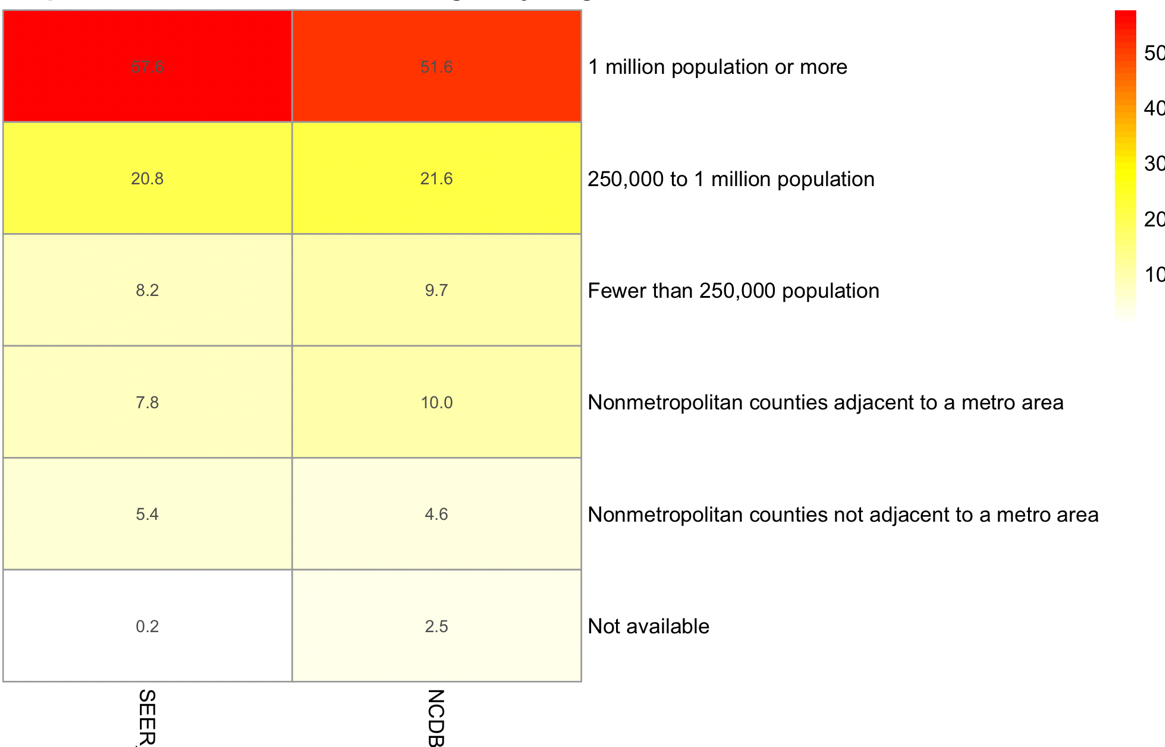


Supplemental Figure 5 Comparison of Area-Based Rurality Measures Across NCDB and SEER Database
